# Self-assembling a 1,4-dioxane-degrading consortium and identifying the key role of *Shinella* sp. through dilution-to-extinction and reculturing

**DOI:** 10.1128/spectrum.01787-23

**Published:** 2023-10-26

**Authors:** Kun Tian, Yue Zhang, Ruihuan Chen, Ding Tan, Ming Zhong, Dandan Yao, Yuanhua Dong, Yun Liu

**Affiliations:** 1 Key Laboratory of Soil Environment and Pollution Remediation, Institute of Soil Science, Chinese Academy of Sciences, Nanjing, China; 2 University of Chinese Academy of Sciences, Beijing, China; 3 University of Chinese Academy of Sciences, Nanjing, China; 4 College of Environment, Hohai University, Nanjing, China; 5 College of Life and Environmental Science, Wenzhou University, Wenzhou, China; Migal-Galilee Research Institute, Kiryat Shmona, Israel

**Keywords:** dilution-to-extinction, next-generation sequencing, consortium, 1,4-dioxane, key degraders

## Abstract

Assembling a functional consortium and identifying novel degraders from contaminated environments are still challenging due to the large diversity of microorganisms and the difficulty in isolating pure cultures. Here, we constructed a relatively simple functional consortium by enriching 1,4-dioxane-degrading-consortia using a culture-dependent dilution-to-extinction (DTE) method and reported a new key dioxane-degrader *Shinella* sp. Our results showed that serial dilution and reculture led to a divergence in the degradation ability of each consortium. Next-generation sequencing data revealed that the divergence in degradation performance was due to the reassembly of microbiota in the DTE process, which occurred most notably in 10^−8^ and 10^−9^ dilutions. The shift in community structure at 10^−9^ prevented the recovery of 1,4-dioxane degradation capacity, and the newly dominant taxa, *Xanthobacter* and *Acinetobacter*, struggled to replace the original dominant genus *Shinella* for 1,4-dioxane biodegradation. Combining differential analysis of community structure and metabolic function, we confirmed that *Shinella* species have a stronger 1,4-dioxane degradation ability than *Xanthobacter* species in the enriched consortium. In addition, we verified our findings using our isolated dioxane-degrading bacteria, *Shinella yambaruensis*, resulting in the rapid recovery of degradation performance of a 10^−9^ dilution consortium with *Xanthobacter* and *Acinetobacter* as the dominant microbiota. Taken together, this study provides a strategy for self-assembling functional consortiums and identifying the key degraders to explore the underlying biological mechanisms of enriched contaminant-degrading consortia.

**IMPORTANCE:**

Assembling a functional microbial consortium and identifying key degraders involved in the degradation of 1,4-dioxane are crucial for the design of synergistic consortia used in enhancing the bioremediation of 1,4-dioxane-contaminated sites. However, due to the vast diversity of microbes, assembling a functional consortium and identifying novel degraders through a simple method remain a challenge. In this study, we reassembled 1,4-dioxane-degrading microbial consortia using a simple and easy-to-operate method by combining dilution-to-extinction and reculture techniques. We combined differential analysis of community structure and metabolic function and confirmed that Shinella species have a stronger 1,4-dioxane degradation ability than Xanthobacter species in the enriched consortium. In addition, a new dioxane-degrading bacterium was isolated, *Shinella yambaruensis*, which verified our findings. These results demonstrate that DTE and reculture techniques can be used beyond diversity reduction to assemble functional microbial communities, particularly to identify key degraders in contaminant-degrading consortia.

## INTRODUCTION

Over the past few years, there has been growing extensive contamination of aquatic environments by the industrial compound 1,4-dioxane (1, 2). This compound is highly soluble (miscible in water) and possesses low adsorption potential (log Koc = 1.23) due to its two ether bonds, allowing for easy entry into water and resulting in large pollution plumes. The US EPA has classified it as a class 2B potential carcinogen, while the European REACH has classified 1B carcinogen (3, 4). As a result, its threat to public water safety cannot be ignored (5, 6). Physical, chemical, and biological methods have been used for 1,4-dioxane treatment, while biological methods have been widely studied and applied for 1,4-dioxane due to their green, low-cost, and non-secondary pollution advantages (7). Both bacteria and fungi have been reported as 1,4-dioxane degraders (8 – 11) through metabolism and co-metabolism (12, 13). Microbial consortia are more effective than pure culture strains, as they possess stronger environmental stress adaptability, richer metabolic pathways, and stronger degradation ability (14 – 16). Recent studies have reported the use of microbial consortia for 1,4-dioxane biodegradation (17 – 19). However, due to the enormous diversity of microbes, assembling a functional consortium and identifying novel degraders for 1,4-dioxane through a simple method remain challenging. To date, research on 1,4-dioxane biodegradation has focused on isolating and characterizing consortium communities (20, 21).

Functional consortia that degrade 1,4-dioxane are typically enriched with 1,4-dioxane as their sole carbon and energy source (17, 21). These consortia exhibited exceptional degradation properties and environmental stress tolerance, attributes believed to arise from the interactions between different microbes within the community (22, 23). As a result, an increasing number of investigations have focused on identifying microbial interactions and keystone taxa within the community (24 – 26). Culture-independent approaches, such as next-generation sequencing (NGS) (27, 28) and meta-omics technologies, coupled with bioinformatics analyses, are commonly used to analyze microbiome data and speculate on intermicrobial interactions. Identification of the core microbiota and predominant species enables the inference of microbial interactions and keystone taxa through correlations and co-occurrence network analysis (24, 29, 30). However, pure culture isolation remains a crucial culture-dependent approach to validate these findings (31). Although effective targets need to be culturable on solid media, pure culture isolation may yield misleading results if it fails to capture the effect targets present in the community. Therefore, it is recommended to adopt a hybrid strategy that combines both culture-dependent and culture-independent methods to accurately identify key degraders in functional consortia.

The dilution-to-extinction (DTE) is a simple and easy-to-operate culture-dependent technique that preserves functional microbial populations of interest (32, 33). For instance, Diaz-Garcia et al. (32) used a combination of dilution-to-stimulation and dilution-to-extinction to construct a minimal yet effective lignocellulolytic bacterial consortium sourced from forest soils. While DTE is frequently applied directly to water, soil, and marine samples (34, 35), it is rarely used to investigate enriched contaminant-degrading bacterial consortia. Therefore, our knowledge of how enriched contaminant-degrading consortia respond to the DTE process is limited.

Using a culture-dependent DTE approach, we hypothesized that reassembly of communities following serial dilution and re-culture with 1,4-dioxane as the sole carbon source would lead to the restoration of key degraders’ predominance. However, the efficiency of the consortia may decline with increasing dilution factors due to the restructuring of the communities and loss of key degraders. Therefore, by comparing different consortia after reassembly, we can identify the essential degraders in the consortia.

In this study, we utilized the DTE method to reconstruct the microbial communities responsible for the degradation of 1,4-dioxane at varying dilution factors. We then employed culture-independent next-generation sequencing to analyze the microbial community dynamics and compared consortia with varying degradation capacities, to identify potential key degraders. To verify the accuracy of our finding, we added the isolated key degraders to poor-performing consortium and confirmed the key members in the degradation of 1,4-dioxane.

## RESULTS

### 1,4-Dioxane degradation in different consortia obtained by DTE

**Fig 1:**
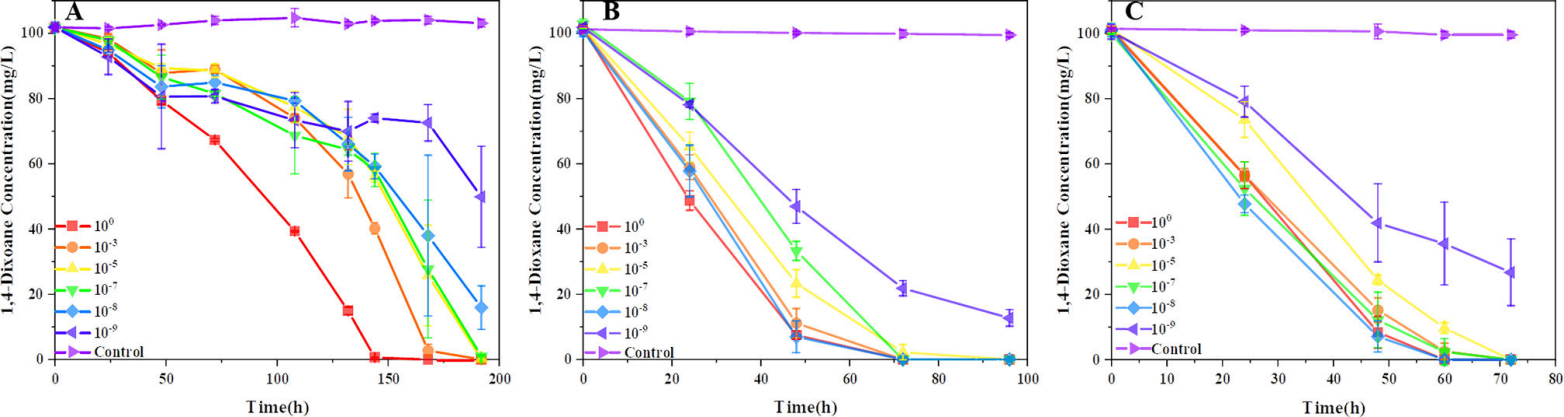
Degradation of 1,4-dioxane by the consortium at different dilution factors (10^0^, 10^−3^, 10^−5^, 10^−7^, 10^−8^, and 10^−9^). (**A**) First inoculation to 100 mg L^−1^ 1,4-dioxane mineral salt medium after serial dilution. (**B**) Second inoculation to 100 mg L^−1^ dioxane. The end solutions of the first degradation (192 hr) were collected as bacterial stock solutions. (**C**)The third inoculation to 100 mg L^−1^ 1,4-dioxane. The end solutions of the second degradation (96 hr) was used as stock solution. Sterilized cells were used as a control group. The error bars indicate the standard deviations (SDs) of three replicates.

To test our hypothesis, we evaluated the ability of each consortium to degrade 1,4-dioxane at different dilutions and performed a total of three inoculations for reculture (Fig. 1). The 10^0^, 10^−3^, 10^−5^, and 10^−7^ dilutions consortium were able to degrade 1,4-dioxane to below the detection limit (1 mg L^−1^) at around 192 h. However, there was a notable difference for the 10^−8^ and 10^−9^ dilution consortia, with residual 1,4-dioxane concentrations of 15.9 ± 6.7 mg L^−1^ and 49.9 ± 15.5 mg L^−1^, respectively (Fig. 1A). We collected cells for a second round of inoculation to assess the recovery and stability of the metabolic function and bacterial consortium during the recultivation. In the second inoculation, the 10^−8^ consortium’s 1,4-dioxane degradation capability recovered rapidly, while the 10^−9^ consortium showed functional differentiation. After the second and third incubations, the 10^−9^ consortium still degrade 100 mg L^−1^ of 1,4-dioxane slowly, with residual concentrations of 12.7 ± 2.5 mg L^−1^ and 26.8 ± 10.2 mg L^−1^ at the end of the process, respectively (Fig. 1B and C). In addition, a clear phenotypic difference was observed between the 10^−9^ consortium and the others, as there was no flocculent formation in the 10^−9^ consortium (Fig. S1). The 10^−9^ consortium’s 1,4-dioxane degradation performance and phenotype distinctly differed from those of other consortia, particularly between the 10^−8^ and 10^−9^ dilutions.

### Microbial community analysis of different consortium

The impact of the DTE method on microbial community composition was evaluated by analyzing 16S rRNA sequencing data. Following enrichment with 1,4-dioxane and magnetic nanoparticle-mediated isolation, only a limited number of microbiota were retained, resulting in a small number of observed species (53) (Table S1). Interestingly, despite serial dilution and reculture of the consortium, the alpha diversity was not significantly affected, as demonstrated by the absence of significant changes in the observed species index (Kruskal-Wallis test, *P* > 0.05) (Table S1). This finding may be attributed to the symbiotic interaction between bacteria in the enriched consortium, as suggested by SEM images of the original consortium and the 10^−9^ consortium (Fig. S2). By contrast, the number of culturable microbial species decreased with serial dilution when using the pure culture-based coating method for both R2A (Fig. S3) and dioxane mineral salt medium (MSM) agar plate (Fig. S4).

**Fig 2:**
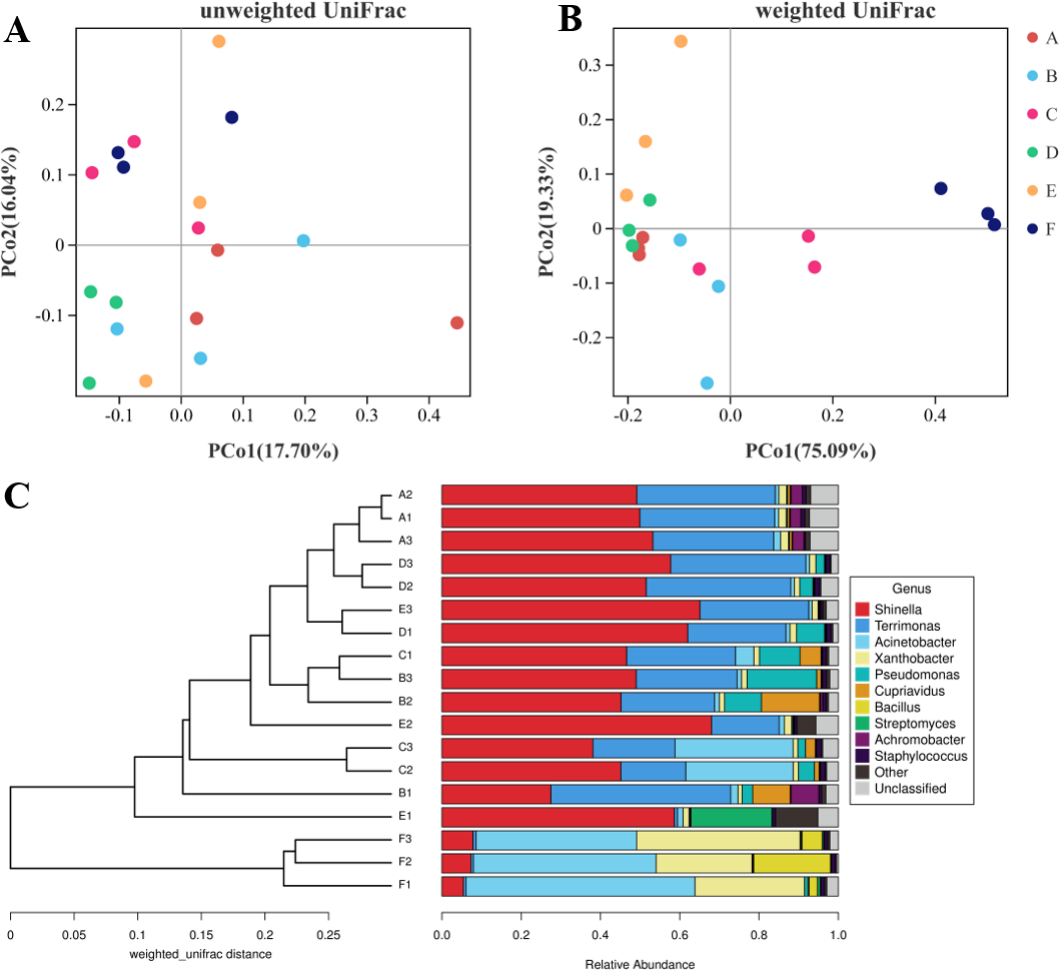
PCOA plots based on UniFrac distance [unweighted (**A**) and weighted (**B**)]. Species compositions are more similar when the samples are closer together. UPGMA clustering analysis based on weighted UniFrac distance and relative abundance of the top 10 dominant genera (**C**). A to F represent the consortia at different dilution factors: 10^0^, 10^−3^, 10^−5^, 10^−7^, 10^−8^, and 10^−9^.

The unweighted UniFrac analysis (PERMANOVA *R* = 0.386, *P* = 0.002) demonstrated that the PCO1-axis accounted for 17.70% of the variability, whereas the PCO2-axis accounted for 16.04% (Fig. 2A). By contrast, the weighted UniFrac distance (PERMANOVA *R* = 0.865, *P* = 0.001) revealed that the PCO1-axis and PCO2-axis could be explained by 75.09% and 19.33% of the total variance, respectively (Fig. 2B). The weighted UniFrac distance analysis was more informative and indicated that there was a shift in the relative abundance of species within the different consortium. Unlike the unweighted UniFrac distance that considered only the presence and absence of species, the weighted UniFrac distance incorporated species abundance information (36). The principal coordinate analysis (PCoA) plot, based on UniFrac distance (unweighted and weighted) (Fig. 2A and B), revealed significant changes in the bacterial community structure due to the DTE approach. This observation suggested that the microbiota underwent reassembly during the DTE and reculture process. The unweighted pair group method with arithmetic mean (UPGMA) dendrogram was constructed using the weighted unifrac distance, and it effectively separated the 10^−9^ dilution from the other consortia (Fig. 2C). This separation of the 10^−9^ consortium on the community structure PCO1 could be linked to the 1,4-dioxane metabolic function divergence during the microbiota reassembly process.

The taxonomic analysis revealed that phylum Proteobacteria was predominant across all dilutions (Fig. S5). In the 10^−9^ consortium, operational taxonomic units (OTUs) assigned to Proteobacteria and Firmicutes exhibited a substantial increase, accounting for 87.81% and 9.99%, respectively (Fig. S5A). Conversely, Bacteroidetes demonstrated a significant decline in the 10^−9^ consortium, comprising only 0.79% (Kruskal-Wallis test, *P* < 0.05). Rhizobiales was the dominant order in all dilutions, with Rhizobiaceae and Xanthobacteraceae as the primary families (Fig. S6A). However, the relative abundance of Rhizobiales carrying Rhizobiaceae significantly decreased in the 10^−9^ consortium, while Xanthobacteraceae exhibited the opposite trend (Fig. S5B and S6A). In addition, the relative abundance of Pseudomonadales at the family level significantly increased to 48.56% (Kruskal-Wallis test, *P* < 0.05) (Fig. S6A). Rhizobiaceae and Chitinophagaceae were replaced by Moraxellaceae and Xanthobacteraceae at the family level in the 10^−9^ dilution consortium (Fig. S6A). At the genus level, *Shinella* and *Terrimonas* were the most abundant genera in all dilutions, except for the 10^−9^ dilution, where the dominant genus shifted to *Acinetobacter* and *Xanthobacter* (Fig. S6B). These genera accounted for 48.11% ± 8.84% and 31.02% ± 9.02% of the microbiota, respectively. The relative composition of bacteria was found to have changed during the dilution and re-culture process, as confirmed by the fact that genera *Shinella* and *Terrimonas* accounted for only 6.86% ± 1.26% in the 10^−9^ dilutions and 0.69% ± 0.10%. Based on a previous study that defined the core microbiota (37), it was found that only *Shinella* and *Xanthobacter* genera exhibited a relative abundance greater than 1% in all dilution samples and were thus classified as the core microbiota of the microbial consortium (Fig. S6B). It is noteworthy that alterations in the relative abundance of core microbiota consistently correlated with changes in degradation function. Furthermore, the linear discriminative analysis (LDA) effect size (LEfSe) analysis revealed 21 biomarkers that could potentially drive metabolic function divergence between different dilutions (Fig. S7). Notably, the Rhizobiaceae family and *Shinella* genus carried by the order Rhizobiales, the *Xanthobacter* genus carried by the Xanthobacteraceae family, and the *Acidetobacter* genus and Moraxellaceae family carried by the order Pseudomonadales were potential biomarkers of functional divergence between the 10^−8^ and 10^−9^ dilution consortia include.

### Genera-to-genera and genera-to-function correlations

**Fig 3:**
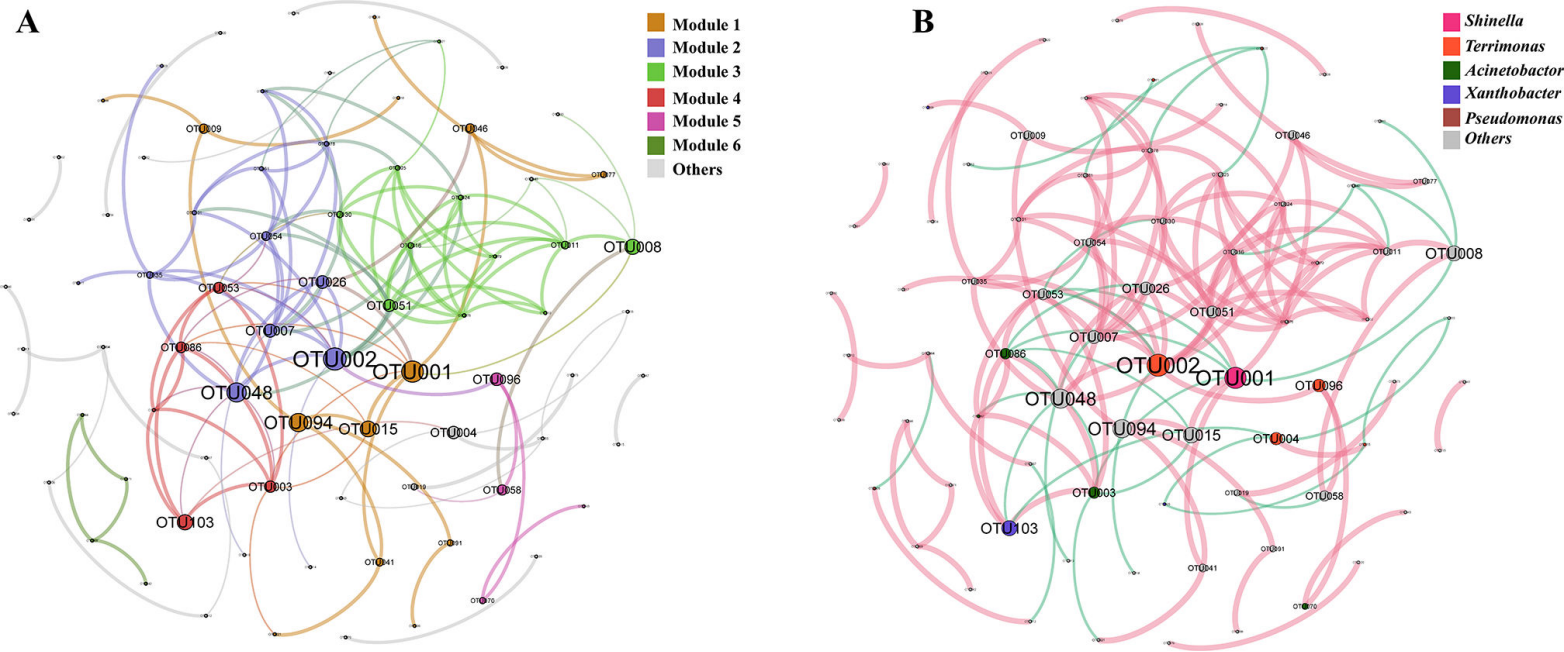
Co-occurrence network analysis of DTE and reculture process. The size of the nodes is proportional to the BC of nodes in networks. (**A)** Colored by modules, six main modules (>5%), and others are classified as others. (**B)** Colored by genus, the top five genera in average relative abundance were colored and the rest were classified as others. Green and red edges indicate positive and negative correlations, respectively. *P*-value < 0.01, the correlation coefficient *r* > 0.6 was considered to be a robust correlation in this DTE process (29, 41).

To investigate the interactions between different microbial taxa in bacterial consortia, we conducted a co-occurrence network analysis based on Spearman’s correlation (Fig. 3). All OTUs were classified into peripherals (*Zi* <2.5 and *Pi* <0.62), indicating that connections mainly occur within the module (38). Betweenness centrality (BC) was also used and calculated in Gephi to evaluate the impact of nodes on other nodes in the network and to identify the centrality of different OTUs in the co-occurrence network (39, 40). The co-occurrence network consisted of 78 nodes and 139 edges, with the OTUs, *Shinella* (OTU1), and *Terrimonas* (OTU2), having the highest BC value. At the genus level (Fig. S8), the predominant genus *Shinella* showed a significant negative correlation with the genera *Acinetobacter* (*P* < 0.01). Meanwhile, the genus *Acinetobacter* also exhibited a significant negative correlation with *Terrimonas* (*P* < 0.01). This suggested that the influence of *Acinetobacter* prevented the genera *Shinella* and *Terrimonas* from becoming the dominant species again, and their utilization ability failed to recover even after multiple inoculations in 1,4-dioxane. By contrast, *Xanthobacter*, a previously identified 1,4-dioxane degraders, was significantly negatively correlated with the genera *Pseudomonas* (*P* < 0.001) and not significantly correlated with the genus *Shinella* (*P* > 0.05).

To investigate the changes in the functional diversity of microbial consortia in response to serial dilutions and reculture, we employed the PICRUSt2 tool to predict the KEGG ortholog (KO) functional changes based on 16S rRNA gene sequence data (42). We focused on pathways related to organic pollutant degradation and found that pathways such as xenobiotics biodegradation, metabolism, membrane transport, lipid metabolism, energy metabolism, transport, and catabolism were significantly enriched in groups 10^−8^ (Kruskal-Wallis test; *P* < 0.05) (Fig. S9). Further inter-group comparison revealed that the 10^−8^ dilution exhibited significantly higher activity than the 10^−9^ across all these functions (Welch’s *t*-test; *P* < 0.05). To emphasize the functional divergence of the microbial consortia in 1,4-dioxane degradation, we selected genes encoding monooxygenase, aldehyde dehydrogenase, and alcohol dehydrogenase, which were closely related to 1,4-dioxane degradation, and identified seven monooxygenases that potentially involved in the metabolism of 1,4-dioxane that were enriched in 10^−8^ dilution (Table S2). These included nitronate monooxygenase, 4-hydroxyphenylacetate 3-monooxygenase, p-hydroxybenzoate 3-monooxygenase, cytochrome P450 monooxygenase, phenol 2-monooxygenase, flavin-dependent monooxygenase, toluene monooxygenase system, and unspecific monooxygenase (K00493).

To further explore the link between the dominant microbiota and the function of 1,4-dioxane degradation, we conducted a Spearman’s rank correlation analysis between the essential enzyme-encoded functional genes and the dominant microbiota at the genus level (Fig. S10). Our analysis revealed that the dominant genus, *Shinella*, exhibited a significantly positive correlation with genes encoding p-hydroxybenzoate 3-monooxygenase, 4-hydroxyphenylacetate 3-monooxygenase, phenol 2-monooxygenase, and unspecific monooxygenase (K00493), but a significantly negative correlation with alkane 1-monooxygenase and 2,4-dichlorophenol 6-monooxygenase. By contrast, *Acinetobacter* showed a significantly positive correlation with alkane 1-monooxygenase and 2,4-dichlorophenol 6-monooxygenase, but a negative correlation with p-hydroxybenzoate 3-monooxygenase and 4-hydroxyphenylacetate 3-monooxygenase. In addition, *Xanthobacter* and *Streptomyces* showed a significantly positive correlation with cytochrome P450 monooxygenase.

### Species and functional profiles comparative analyses of 10^−8^ and 10^−9^ dilutions

To investigate the effects of DTE-driven microbiota reassembly on the taxonomy and functional gene level diversity, we conducted a metagenomic analysis on 10^−8^ and 10^−9^ dilutions. The Rhizobiales order was found to be the dominant microbiota in both consortia, with relative abundance of 81.33% and 73.20%, respectively (Fig. S11A). However, we observed significant differences at the family level, with the Rhizobiaceae family accounting for 59.89% in the 10^−8^ consortium but only 2.87% in the 10^−9^ consortium. By contrast, the Xanthobacteraceae family accounted for 60.47% in the 10^−9^ consortium, but only 0.15% in the 10^−8^ consortium (Fig. S11B). LEfSe analysis confirmed that Rhizobiaceae carrying the genera *Shinella*, *Rhizobium,* and *unclassified_Rhizobiales* were biomarkers of the 10^−8^ consortium, while the 10^−9^ included Xanthobacteraceae carrying the genera *Xanthobacter* and Moraxellaceae carrying the genera *Acinetobacter* (Fig. S12). Overall, our findings suggested that DTE-induced reassembly of microbiota could lead to significant shifts in taxonomic and functional gene diversity, particularly at the family level.

**Fig 4:**
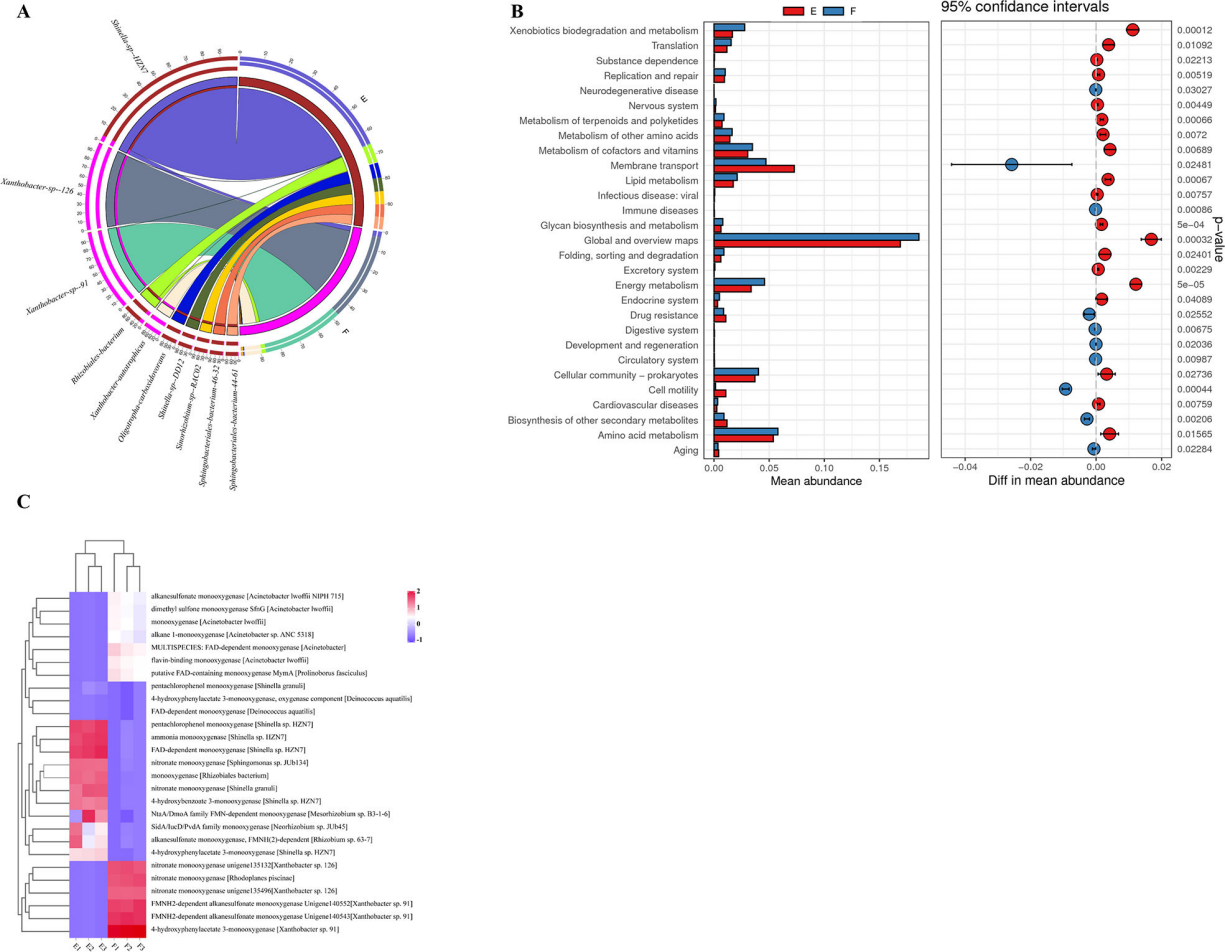
Metagenomic study of microbial consortia 10^−8^ (E) and 10^−9^ (F). (**A**) Top 20 species annotated in metagenomics. (**B**) Welch’s *t*-test of KEGG metadata categories Level 3 (*P* < 0.05). Of which 29 pathways were demonstrated with significant differences. (**C**) Monooxygenase-encoding genes with significant difference (*P* < 0.05).

The relative abundance of the genera *Shinella* was significantly higher in the 10^−8^ consortium (44.96% ± 8.87%) compared to the 10^−9^ consortium (1.61% ± 2.68%) (Fig. S13). Within the 10^−8^ consortium, *Shinella*_sp_HZN7 was the most abundant species, representing 17.97%–25.13%, whereas in the 10^−9^ consortium, it only accounted for 0.02%–2.32% (Fig. 4A; Fig. S14). *Rhizobiales*_bacterium had a higher relative abundance in the 10^−8^ consortium (3.08% ± 0.38%) than in the 10^−9^ consortium (1.11% ± 0.13%). Similarly, the relative abundance of genus *Rhizobium* was higher in the 10^−8^ consortium (4.07% ± 1.07%) than in the 10^−9^ consortium (0.53% ± 0.17%). By contrast, the genera *Xanthobacter* were more prevalent in the 10^−9^ consortium (58.59% ± 3.27%) compared to the 10^−8^ consortium (0.1% ± 0.5%). Within the 10^−9^ consortium, *Xanthobacter*_sp_126 and *Xanthobacter*_sp_91 accounted for 12.19% ± 0.65% and 11.23% ± 0.59%, respectively.

The unigenes were subjected to annotation in KO groups and were further assigned to specific KEGG metabolic pathways (43). The KEGG annotation results revealed that the majority of genes in the 10^−8^ and 10^−9^ bacterial consortia were located in the metabolism at KEGG Level 1 (Fig. S15). Among all the enriched KEGG pathways, 21,529 genes (58.64%) were assigned to metabolic pathways (ko01100) and 6,881 genes (18.74%) were assigned to microbial metabolism in diverse environments (ko01120). Genes involved in global and overview maps, metabolism of carbohydrates, and metabolism of amino acids were the most abundant. Interestingly, the distribution of annotated unigenes assigned to the KEGG pathway between consortia 10^−8^ and 10^−9^ (Fig. 4B; Fig. S16) appeared to be opposite to the predicted functional profiles generated by PICRUSt2. The 10^−8^ and 10^−9^ consortia exhibited significant differences in the xenobiotics biodegradation and metabolism, global and overview maps, energy metabolism, lipid metabolism, and amino acid metabolism pathways, all above link to Level 1 metabolism. Moreover, the 10^−8^ consortium was lower in abundance than the 10^−9^ consortium in all aspects except membrane transport. These unexpected trends between metagenome and PICRUSt2-predicted data in the KEGG functional pathway could be attributed to the discrepancy between the depths of 16S rRNA and metagenomic sequencing. Subsequently, we conducted DESeq2 analysis to identify the genes expressed in the microbiome data. Notably, a total of 13,623 genes were significantly upregulated in the 10^−8^ consortium, while 51,838 genes were significantly downregulated in the 10^−8^ consortium (Fig. S17).

To compare the ability of the 10^−8^ and 10^−9^ consortia to degrade 1,4-dioxane, we analyzed the genes involved in the degradation process. Specifically, we focused on genes encoding monooxygenase enzymes and their corresponding species information in the NCBI-NR database (Fig. 4C; Table S3). A total of 39 monooxygenase-encoding genes were identified that were potentially involved in contaminant metabolism and significantly different between the two consortia (*P* < 0.05). *Rhizobiales* bacterium, carrying monooxygenases such as *Shinella* sp. HZN7 with 4-hydroxybenzoate 3-monooxygenase, FAD-dependent monooxygenase, and 4-hydroxyphenylacetate 3-monooxygenas, as well as pentachlorophenol monooxygenase, may be strongly linked to the superior 1,4-dioxane degradation ability of the 10^−8^ consortium (Fig. 4C). By contrast, *Acinetobacter* and *Xanthobacter* species carrying genes encoding monooxygenases exhibited weaker degradation capacity for 1,4-dioxane. These findings were consistent with the observed species composition differences between the 10^−8^ and 10^−9^ consortia. These results support the idea that the functional divergence was due to the reassembly of the dominant microbiota in the consortium. In addition, our results highlighted the important metabolic functions of *Shinella* species in 1,4-dioxane degradation, which were dominant in each group except 10^−9^.

### Recovery of degradation capability through the *Shinella* sp. isolate

**Fig 5:**
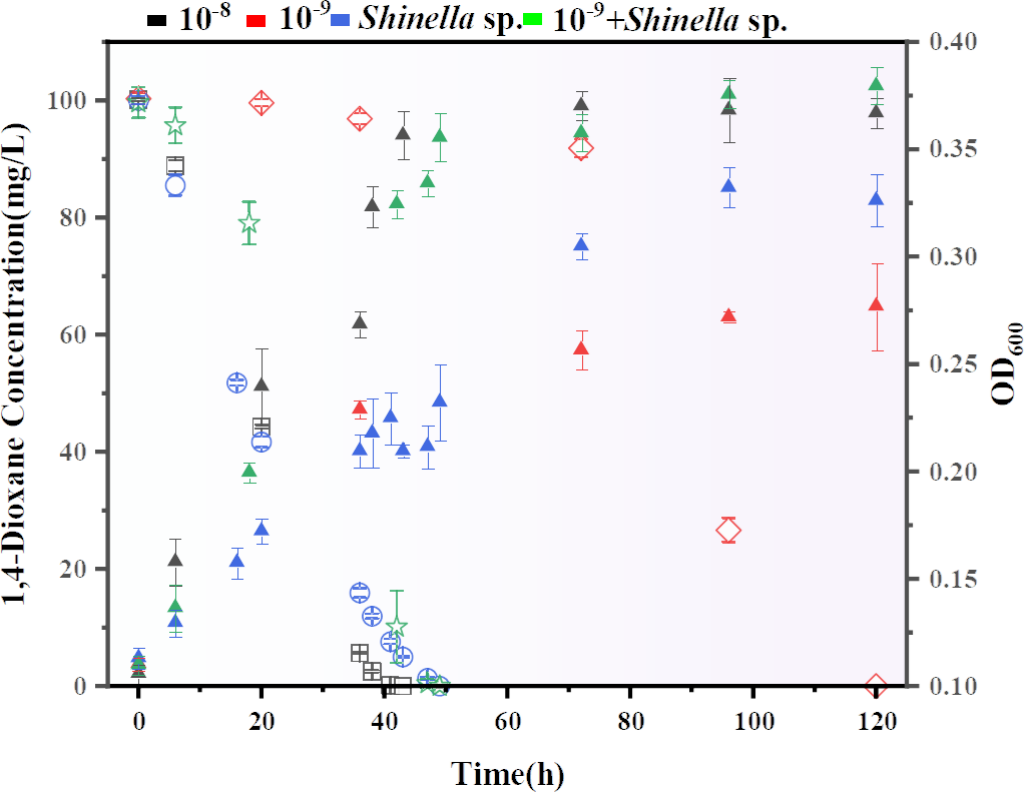
1,4-Dioxane degradation performance of 10^−8^, 10^−9^ consortium, and 10^−9^ after adding DXTK-001 (*Shinella yambaruensis* strain). All experiments were conducted at an initial OD_600_ of 0.1. C_0_ = 100 mg L^−1^, *n* = 3.

Finally, additional experiments were conducted to isolate a pure culture of *Shinella* species and explore its capability to degrade 1,4-dioxane (Fig. 5; Fig. S18). Only one of the 182 OTUs identified belongs to the *Shinella* genera, with a sequence that showed 99.09% maximum identity with *Shinella yambaruensis* (NR_114035.1). To validate the pivotal role of *Shinella* species on the degradation of 1,4-dioxane with the microbial consortia, a pure culture named DXTK-001 was isolated from the consortium. This pure culture had 100% maximum identity with *Shinella yambaruensis* (NR_114035.1) and was added to the 10^−9^ consortium (Fig. S19). The results demonstrated a rapid restoration of strong 1,4-dioxane degradation ability in the microbial consortia after the addition of *Shinella yambaruensis* to the 10^−9^ consortium (Fig. 5). Furthermore, compared to the 10^−8^ consortium, the 10^−9^ microbial consortium reused as a stock solution for inoculation at 100 mg L^−1^ was less effective in degrading 1,4-dioxane than before, with limited degradation occurring in the first 3 days. At a concentration of 200 mg L^−1^, the same results were observed, with degradation completed in about 20 days for the 10^−9^ consortium (Fig. S20). These results strongly support the vital role of *Shinella* species in the degradation of 1,4-dioxane. Despite our initial assumption that *Xanthobacter* was the primary degrader (18), the degradation of 1,4-dioxane was relatively slow in the 10^−9^ consortium, which had a high abundance of *Xanthobacter* species. By contrast, *Shinella* species were detected at a lower abundance in the 10^−9^ dilutions.

## DISCUSSION

Top-down enrichment using contaminants as the sole carbon and energy source is a common method for obtaining functional microbial populations. However, the resulting bacterial consortia can contain hundreds of different species, making it challenging to identify key degraders. To overcome this issue, the DTE method was often used to reduce microbial diversity and capture functional microbiota from environmental samples such as soil and ocean (44 – 47). The DTE method also maximized the self-assembling properties of the microbiota (45). Instead of applying the DTE method to raw environmental samples, we reassembled the enriched consortium during serial dilution and reinoculation to differentiate key degraders and functional genes by comparison of functional profile and microbial community using culture-dependent DTE and culture-independent NGS method. Our aim was to extend the DTE method to degrading bacterial consortia, to identify key degraders and functional genes.

We utilized the DTE method on a microbial consortium capable of degrading 1,4-dioxane, which was enriched from 0 to 1,000 mg L^−1^ 1,4-dioxane selective pressure, and obtained metabolic microbiota using magnetic nanoparticle-mediated isolation (18). After serial dilution, we selected the 10^−3^, 10^−5^, 10^−7^, 10^−8^, and 10^−9^ dilutions to study the changes in 1,4-dioxane degradation capabilities. When re-inoculated into MSM medium with 100 mg L^−1^ 1,4-dioxane as the sole carbon source, the degradation efficiency diverged, particularly between 10^−8^ and 10^−9^ (Fig. 1). The 10^−9^ dilution consortium was unable to restore its initial degradability toward 1,4-dioxane, while the 10^−8^ dilution had recovered. We speculate that the DTE and reculture process resulted in a change in the dominant microbiota, which was more evident between 10^−8^ and 10^−9^ dilutions, and verify microbial communities and identify the key degraders using NGS technology.

The DTE method, combined with reculture, did not significantly alter alpha diversity in the microbial consortia (Table S1). This is likely due to the top-down enrichment and MMI process, which promote symbiosis in the consortium. However, beta diversity analysis showed significant differences in community structure (PERMANOVA *R* values = 0.864, *P* = 0.001), with the most pronounced changes occurring in the 10^−9^ dilution. The divergence in community structure of the 10^−9^ dilution consortium was consistent with the degradation capacity observed. By contrast, reculture in liquid medium was not possible using the pour plate method on R2A and MSM solid medium (Fig. S3 and S4). This is because the agar solid medium is still based on culturable microorganisms in the consortia. The high symbiotic capacity of bacteria in the consortium resulted in a lesser impact on community alpha diversity by the DTE method, indicating that DTE provides more information than directly isolated degraders on agar plates. The phylum Proteobacteria carrying order Rhizobiales remained the most dominant microbiota in all consortia, owing to their adaptation and utilization towards 1,4-dioxane (48, 49) (Fig. S5). Within Rhizobiales, the genera *Shinella* and *Xanthobacter* were the core microbiota, with relative abundances greater than 1% in all dilution samples (Fig. S6B). However, the dominance of the genera *Shinella* and *Terrimonas* started to shift at 10^−9^ dilution, with *Acineobacter* and *Xanthobacter* becoming the dominant microbiota. Thus, the divergence in the 10^−9^ dilution degradation capability can be attributed to the reassembly of the dominant microbiota. The original dominant populations, *Shinella* and *Terrimonas*, were constrained in 10^−9^ and did not regain degradability in all three degradation cultures. By contrast, microbiota reassembly in 10^−8^ dilutions allowed *Shinella* to become the most predominant genus (63.92%) and rapidly regained its degradation capacity. LEfSe analysis further identified potential biomarkers *Shinella*, *Xanthobacter*, and *Acineobacter* for degradation functional divergence in 10^−8^ and 10^−9^ (Fig. S7).

We used the PICRUSt2 functional prediction tools to analyze the functional changes that occurred during the DTE processing. KEGG pathways associated with 1,4-dioxane biodegradation were found to be enriched in the 10^−8^ dilution, including pathways related to xenobiotics biodegradation and metabolism, metabolism of other amino acids, lipid metabolism, energy metabolism, and transport and catabolism (Fig. S9). Furthermore, we identified the functional genes that directly contributed to 1,4-dioxane biodegradation, such as monooxygenase, alcohol dehydrogenase, and aldehyde dehydrogenase in the KO database (Tables S3, S5, and S6). Nitronate monooxygenase, 4-hydroxyphenylacetate3-monooxygenase, p-hydroxybenzoate3-monooxygenase, cytochrome P450 monooxygenase, and phenol 2-monooxygenase were all significantly enriched in 10^−8^ dilution. The differentiation in composition between 10^−8^ and 10^−9^ dilutions resulted in significant changes in their microbial community function, as evidenced by the weighted UniFrac distance matrix (Fig. 2A) and PICRUSt2 function prediction (Fig. 2B; Fig. S9). Importantly, the reassembly of the microbiota during dilution and reculture resulted in changes in 1,4-dioxane metabolic function, particularly between 10^−8^ and 10^−9^ dilutions.

Understanding the interactions between microbial species in a consortium is crucial for comprehending community reassembly during DTE treatment. Our co-occurrence network analysis revealed that OTU1 (*Shinella*) and OTU2 (*Terrimonas*) were situated at the core of the network with the highest BC values. The lower BC values of OTU3 (*Acinetobacter*) and OTU5 (*Xanthobacter*), which predominated the dominant microbiota in the 10^−9^ consortium, implied their more peripheral position in the co-occurrence network (Table S4). The observed divergence in the degradability of the 10^−9^ consortium was consistent with this community composition. The significantly negative correlation between *Shinella* and *Acinetobacter*, as well as between *Terrimonas* and *Acinetobacter*, may prevent the genera *Shinella* and *Acinetobacter* from regaining dominance (Fig. S8). These interactions suggested that species interactions played a significant role in the 1,4-dixoane degradation function of consortia. The correlation between the dominant microbiota and the functional genes involved in 1,4-dioxane degradation, as predicted by PICRUSt2, can be used to demonstrate the degradation ability of the dominant taxa. The genera *Shinella* was significantly positively correlated with 4-hydroxyphenylacetate 3-monooxygenase, p-hydroxybenzoate 3-monooxygenase, and phenol 2-monooxygenase, but significantly negatively correlated with 1-monooxygenase and 2,4-dichlorophenol 6-monooxygenase. By contrast, the genera *Acinetobacter* was significantly positively correlated with alkane 1-monooxygenase, 2,4-dichlorophenol 6-monooxygenase, but negatively correlated with the others (Fig. S10). The ability to degrade 1,4-dioxane has been reported before for both *Acinetobacter* and *Xanthobacter*, which are the main microbiota in the 10^−9^ consortium (50, 51).

Metagenome-wide analysis was performed on the two functionally divergent 10^−8^ and 10^−9^ consortia, and a Mantel test analysis was used to assess the correlation between 16S rRNA sequence and metagenome data. Both species and function were found to be significantly correlated (Fig. S21 and S22). Metagenomic analysis showed significant differences in dominant species between 10^−8^ and 10^−9^ dilutions (Fig. S14), and LEfSe analysis confirmed the biomarker taxon for the functional divergence between the two groups (Fig. S12). In 10^−8^ dilutions, the dominant microbiota was *Shinella*, accounting for 58.59%, with *Shinella*_sp._HZN7 being the most abundant at 22.46%. By contrast, *Xanthobacter* was the dominant microbiota in 10^−9^ dilutions, accounting for 44.96%, with *Xanthobacter*_sp._126 and *Xanthobacter*_sp._91 being the dominant species, for 12.19% and 11.23%, respectively. PICRUSt2 predicted profiles and metagenome functional profiles exhibited opposite results for Level 1 metabolism between 10^−8^ and 10^−9^ (Fig. S23). This finding suggested that further analysis of specific functional genes involved in 1,4-dioxane degradation was needed. Functional genes such as p-hydroxybenzoate 3-monooxygenase (*Shinella*_sp._HZN7), monooxygenase (*Rhizobiales_bacterium*), FAD-dependent monooxygenase (*Shinella*_sp._HZN7), 4-hydroxyphenylacetate 3-monooxygenase (*Shinella*_sp._HZN7) were enriched in 10^−8^, and these genes may play a key role in 1,4-dioxane degradation. These results support the important role of *Shinella* species in 1,4-dioxane degradation and its status as a key member of the degradation consortia. Furthermore, a strain of *Shinella yambaruensis* that can use 1,4-dioxane as the sole carbon source and completely degrade 1,4-dioxane was isolated, and by adding it to the 10^−9^ dilution, the consortium’s ability to degrade 1,4-dioxane was quickly restored (Fig. 5; Fig S18 and S20).

In summary, this study has broad applicability in differentiating key degraders of contaminant-degrading bacterial consortia. Previous works have focused on the enrichment of specific microbial degradation consortia from different environments with organic contaminants as the sole carbon source, such as polycyclic aromatic hydrocarbons (52), antibiotics (53, 54), and others (55). Although metabolic interactions among microbiota in degrading consortia are relatively complex, an increasing number of recent efforts have combined next-generation sequencing technologies with culture-based techniques to identify key microorganisms (56). Our results support the hypothesis that the DTE method and reculturing of 1,4-dioxane-degrading bacterial consortium leads to the reassembly of the microbial community and highlights the degradation capacity of key microbial taxa. Furthermore, we isolated the key microorganisms for validation and drew conclusions that *Shinella* species played a key role in 1,4-dioxane degradation. We believe that future studies can extend the application of the DTE method for community reassembly of contaminant-degradation microbial consortia to a wider range of community and functional studies.

### Conclusion

In general, the DTE method was used to collect metabolically functional microbiota by reducing the diversity of the raw environmental samples. However, unlike previous studies, our approach was applied to an enriched 1,4-dioxane-degrading microbial consortium, and we observed that the alpha diversity remained relatively unchanged even after dilution to 10^−9^ with reculture. Instead, the reassembly of the community during the DTE and reculture process resulted in significant differences in the dominant microbiota, leading to functional divergence and identification of key degraders. Our results suggested that in addition to culture-independent NGS-based techniques and bioinformatic analysis, the culture-dependent DTE method can provide valuable insights into identifying key degrader. Our findings warrant further research to extend the application of this method to identify key degraders in a broader enriched contaminant-degrading consortium. Thus, the combination of culture-dependent DTE and culture-independent NGS could be a powerful tool for identifying the key degraders in enriched contaminant-degrading bacterial consortia.

## MATERIALS AND METHODS

### Chemicals and analytic method

1,4-Dioxane (anhydrous, 99.8%) and dichloromethane (anhydrous, ≥99.8%) were purchased from Sigma-Aldrich (St. Louis, MO, USA). The components of the MSM and trace elements were consistent with our previous reports (18). All other chemicals used in this study were of analytical purity grade. 1,4-Dioxane was monitored over time using an Agilent 8860 gas chromatograph equipped with a flame ionization detector (GC-FID) (Agilent, Santa Clara, CA, US).

### Functional consortium, dilution-to-extinction, and reculturing methods

The functional microbial consortium employed in this study had previously been enriched from activated sludge in our laboratory using magnetic nanoparticles (18) and is referred to as the “A” group in the text. The active consortium was collected when it degraded 1,4-dioxane (C_0_ = 100 mg L^−1^) to the endpoint of degradation. Serial dilution experiments (10^−1^–10^−9^) were then conducted to obtain a new generation of a consortium with different microbial community compositions. Briefly, 1 mL of bacterial stock solution was mixed with 9 mL of sterilized 0.85% NaCl solution (pH = 7.0), and gradient dilution was performed in sequence. The diluted solution was then mixed with 20 mL MSM to make a final volume of 30 mL, and 100 mg L^−1^ of 1,4-dioxane was added as the sole carbon source. To increase the difference in community structure, we selected the 10^0^, 10^−3^, 10^−5^, 10^−7^, 10^−8^, and 10^−9^ diluted consortia for re-culturing. The first reculture to the endpoint of degradation of the consortium was considered to complete the DTE community reassembling. The endpoint solution was used as a stock solution to inoculate 3% (vol:vol) into 30 mL of 100 mg L^−1^ 1,4-dioxane MSM for the second degradation experiment. A total of three culture degradation experiments were performed, each with three replicates and incubated at 30°C, 150 rpm.

### Bacterial DNA extraction and 16S rRNA gene amplicon sequencing

A 10 mL of the bacterial consortium suspension at the endpoint of the culture experiment was collected and centrifuged at 12,000 rpm for 10 min at 4°C. The detailed DNA extraction and data analysis process are shown in SI

### Metagenomics sequencing

Metagenomics sequencing was conducted on the same DNA samples used for 16S rRNA sequencing. Initially, the genomic DNA was fragmented using sonication to 350 bp. Subsequently, the fragments underwent end-repaired, A-tailed, and adaptor ligation using NEBNext ΜLtra DNA Library Prep Kit for Illumina (NEB, USA), in accordance with the prescribed protocol. DNA fragments measuring between 300 and 400 bp were then enriched *via* PCR and purified using the AMPure XP system (Beckman Coulter, Brea, CA, USA). The libraries were evaluated for size distribution using the 2100 Bioanalyzer (Agilent, Santa Clara, CA, USA) and quantified using real-time PCR. Genome sequencing was performed using the pair-end technology (PE 150) on the Illumina Novaseq 6000 sequencer.

Each sample’s clean reads were independently assembled using MEGAHIT (version 1.1.2) (57). Genes were predicted from contigs that exceeded 500 bp using MetaGeneMark (V2.10, default setting) with the default setting. A non-redundant gene catalog was created using CD-HIT with 95% identity and 90% coverage. The clean reads were aligned with Unigenes employing Bowtie2 (58). Unigenes were annotated utilizing diamond BLAST software against seven public databases: NR, KEGG, CAZy, CFDB, eggNOG, CARD, and PHI. Taxonomic profiles were generated using Kaiju (version 1.6.3) with the NCBI database (59), and clean reads were used in the process.

### Isolation of pure culture bacteria

The 10^−8^ and 10^−9^ consortia were subjected to further dilution in sterilized 0.85% NaCl solution with a pH of 7.0, ranging from 10^−1^ to 10^−9^. The diluted samples were then plated on both R_2_A agar plates and MSM agar plates, supplemented with 200 mg L^−1^ 1,4-dioxane as the sole carbon source. Single colonies were carefully selected, and their identity was determined using 16S rRNA gene sequences by Sanger sequencing. The resulting sequences were submitted to the NCBI BLAST database for blasting (60).

### Statistical analysis

Concentration data for 1,4-dioxane was analyzed using Microsoft Excel 2019. To examine the effect of dilution factors on community structure, PERMANOVA (999 permutations) was implemented by utilizing the adonis2 function from the vegan R package. Potential microbial biomarkers were determined through the use of LEfSe, which can be found at http://huttenhower.sph.harvard.edu/lefse/ (61). Spearman’s rank correlations between the abundance of OTUs were determined using the R package Hmisc. To explore and visualize the network, Gephi software (version 0.9.2) was employed, and betweenness centrality was used to determine the centrality of a node in the network. To perform the PCoA, Welch’s *t*-test, and Wilcoxon rank test, the R project vegan package and base functions were utilized. The R language GUniFrac package was used to calculate the weighted UniFrac distance based on the OTU sequence phylogenetic tree and OTUs abundance table. Finally, data visualization was accomplished using the ggplot2 package in the R project.

## Data Availability

The Illumina sequence data were deposited in the NCBI Sequence Read Archive (SRA) and available through accession number BioProject PRJNA942363. The data sets for metagenomics were available in BioProject PRJNA1016107. The 16S rRNA sequence of the DXTK-001 (Shinella species) strain was deposited into GenBank under OQ592393.1.

## SUPPLEMENTAL MATERIAL

Supplemental material spectrum.01787-23-s0001.pdf Tables S1 to S6 and Fig. S1 to S23.

ASM does not own the copyrights to Supplemental Material that may be linked to, or accessed through, an article. The authors have granted ASM a non-exclusive, world-wide license to publish the Supplemental Material files. Please contact the corresponding author directly for reuse.
