# Supplementary material for: Self-assembling a 1,4-dioxane-degrading consortium and identifying the key role of Shinella sp. through dilution-to-extinction and reculturing
Source: Microbiol Spectr. 2023 Oct 26;11(6):e01787-23. doi: 10.1128/spectrum.01787-23 (PMC10714792; doi:10.1128/spectrum.01787-23)
Supplement: Supplemental material — Tables S1 to S6 and Fig. S1 to S23. [file spectrum.01787-23-s0001.pdf]

Supplemental Material

Number of pages = 28

Number of Tables = 6

Number of Figures = 23

Supplementary Information 1 DNA extraction and PCR amplification methods

Total DNA was extracted using the HiPure Soil DNA Kits (Magen, Guangzhou, China) according to manufacturer's protocols. The 16S rDNA target region of the ribosomal RNA were amplified by PCR. The DNA quality was detected using Qubit (Thermo Fisher Scientific, Waltham, MA) and Nanodrop (Thermo Fisher Scientific, Waltham, MA) accordingly. The primer sequence used in amply 16S rRNA gene at the hypervariable V3-V4 regions in bacteria was: 341F: 5'-CCTACGGGNGGCWGCAG-3'; 806R: 5'-GGACTACHVGGGTATCTAAT-3'. To get high quality clean reads, raw reads were further quality controlled (QC) using FASTP (version 0.18.0). Paired-end clean reads were merged as raw tags using FLSAH (version 1.2.11) use a minimum 10-bp overlap and mismatch error rates of 2%. Noisy sequences of raw tags were filtered by QIIME (version 1.9.1) pipeline under specific filtering conditions to obtain the high-quality clean tags. The effective tags were clustered into operational taxonomic units (OTUs) of $\geq$ 97% similarity using UPARSE (version 9.2.64) pipeline. Representative sequences of each OTU were then categorized into organisms through an RDP-Ⅱ naive Bayesian classifier (version 2.2) based on the SILVA database (version 132) with the confidence threshold value of 0.8.

Table S1. Bacterial alpha diversity of all dilutions' consortium. (A: Original, B:10^{-3}, C:10^{-5}, D:10^{-7}, E:10^{-8}, F:10^{-9}).

Group	Observed_species	Shannon	Simpson	Chao1	Ace	Goods_coverage	Pd_whole_tree
A1	57.000	2.172	0.661	59.000	58.623	0.999	7.269
A2	54.000	2.168	0.666	56.143	58.498	0.999	6.923
A3	49.000	2.109	0.645	49.000	49.297	0.999	6.486
B1	48.000	2.759	0.806	50.000	50.906	0.999	6.025
B2	55.000	2.468	0.735	59.200	59.401	0.999	6.927
B3	56.000	2.109	0.673	78.500	63.454	0.999	6.912
C1	52.000	2.393	0.714	53.500	53.503	0.999	6.514
C2	56.000	2.302	0.706	56.600	57.798	0.999	7.433
C3	49.000	2.427	0.742	51.500	52.644	0.999	6.341
D1	46.000	1.809	0.567	49.750	49.458	0.999	5.289
D2	50.999	2.083	0.664	53.625	55.548	0.999	6.276
D3	50.999	1.800	0.590	58.500	61.315	0.999	6.539
E1	55.000	2.193	0.608	60.000	59.623	0.999	6.992
E2	55.000	1.687	0.505	59.667	61.168	0.999	7.352
E3	52.000	1.708	0.537	57.143	58.258	0.999	6.669
F1	52.000	1.820	0.586	59.200	58.278	0.999	6.314
F2	47.000	2.107	0.694	56.000	59.145	0.999	6.867

F3	53.000	1.968	0.655	60.000	58.424	0.999	6.965

Table S2. PICRUSt2 predicted monooxygenase encoded genes in KEGG Orthology (KO) database that are potentially involved in 1,4-dioxane degradation. The table presents the mean values of gene copies for each group (n=3).

Description	A	B	C	D	E	F
nitronate monooxygenase	113840	95185	116825	116912	152488	149081
4-hydroxyphenylacetate3-monooxygenase	47762	37457	37162	55073	66501	40907
p-hydroxybenzoate3-monooxygenase	46838	38257	36909	54055	79308	7626
unspecific monooxygenase	46574	35087	35643	53203	79336	7309
alkane 1-monooxygenase	1194	4535	18545	2429	1543	45077
2,4-dichlorophenol 6-monooxygenase	1077	953	16799	855	967	44931
cytochrome P450 monooxygenase	115	111	108	117	7169	344
4-hydroxyphenylacetate 3-monooxygenase	69	1794	873	783	52	56
tmoF, tbuC, touF; toluene monooxygenase electron transfer component	87	1035	347	25	50	34
phenol 2-monooxygenase	33	28	30	34	1058	63
flavin-dependent monooxygenase	1	1	1	3	68	9
toluene monooxygenase system	8	6	5	3	27	1

Table S3. Monooxygenase encoded genes that potentially involved in 1,4-dioxane degradation in metagenome data. Only the genes that exhibited significant differences between groups ($P < 0.05$ for Deseq2) and were present in at least two samples were shown.

Description	E1	E2	E3	F1	F2	F3
nitronate monooxygenase [Shinella granuli]	2687	4092	4438	0	347	0
monooxygenase [Rhizobiales bacterium]	2900	3709	4273	0	327	0
4-hydroxybenzoate 3-monooxygenase [Shinella sp. HZN7]	2718	3400	3862	0	348	0
NtaA/DmoA family FMN-dependent monooxygenase [Mesorhizobium sp. B3-1-6]	273	4770	3416	0	0	0
4-hydroxyphenylacetate 3-monooxygenase [Shinella sp. HZN7]	1485	2008	2365	0	174	0
toluene monooxygenase, partial [Enterobacter hormaechei]	168	2007	1188	3070	2672	3220
Toluene-4-monooxygenase system protein D (modular protein) [Bradyrhizobium sp. ORS 285]	146	1931	1272	3071	2665	3420
antibiotic biosynthesis monooxygenase [Shinella zoogloeoides]	810	1108	1163	0	109	0
antibiotic biosynthesis monooxygenase [Shinella sp. DD12]	842	886	1044	0	109	0
alkane 1-monooxygenase [Sphingobacteriales bacterium 46-32]	0	316	349	0	0	0
pentachlorophenol monooxygenase [Shinella granuli]	18	231	185	0	0	0
antibiotic biosynthesis monooxygenase [Emticicia sp. CRIBPO]	0	89	124	0	0	0
antibiotic biosynthesis monooxygenase [Oligotropha carboxidovorans]	44	120	40	0	0	0
antibiotic biosynthesis monooxygenase [Pseudoflavitalea sp. X16]	0	97	112	0	0	0
phenylalanine 4-monooxygenase [Sphingobacteriales bacterium 46-32]	0	87	106	0	0	0
antibiotic biosynthesis monooxygenase [Oligotropha carboxidovorans]	64	93	0	0	0	0

monooxygenase [Nocardia sp. 852002-20019_SCH5090214]	75	1	0	0	0	0
FAD-dependent monooxygenase [Deinococcus aquatilis]	27	28	5	0	0	0
4-hydroxyphenylacetate 3-monooxygenase, oxygenase component [Deinococcus aquatilis]	28	21	6	0	0	0
antibiotic biosynthesis monooxygenase [Deinococcus aquatilis]	25	19	3	0	0	0
antibiotic biosynthesis monooxygenase [Deinococcus aquatilis]	13	17	2	0	0	0
antibiotic biosynthesis monooxygenase [Deinococcus aquatilis]	21	9	0	0	0	0
4-hydroxyphenylacetate 3-monooxygenase [Xanthobacter sp. 91]	0	0	0	7272	6875	8345
FMNH2-dependent alkanesulfonate monooxygenase [Xanthobacter sp. 91]	0	0	0	5863	5475	6185
FMNH2-dependent alkanesulfonate monooxygenase [Xanthobacter sp. 91]	0	0	0	5598	4990	6177
nitronate monooxygenase [Rhodoplanes piscinae]	0	0	0	5184	4838	5711
nitronate monooxygenase [Xanthobacter sp. 126]	0	0	0	5307	4917	5365
nitronate monooxygenase [Xanthobacter sp. 126]	0	0	0	4970	4516	5193
antibiotic biosynthesis monooxygenase [Xanthobacter sp. 91]	0	0	0	2484	2164	2890
MULTISPECIES: FAD-dependent monooxygenase [Acinetobacter]	0	0	0	2831	2189	2012
putative FAD-containing monooxygenase MymA [Prolinoborus fasciculus]	0	0	0	2514	2045	1770
flavin-binding monooxygenase [Acinetobacter lwoffii]	0	0	0	2287	1944	1839
antibiotic biosynthesis monooxygenase [Xanthobacter sp. 91]	0	0	0	1813	1663	1963
alkanesulfonate monooxygenase [Acinetobacter lwoffii NIPH 715]	0	0	0	2118	1763	1546
monooxygenase [Acinetobacter lwoffii]	0	0	0	2034	1812	1465
dimethyl sulfone monooxygenase SfnG [Acinetobacter lwoffii]	0	0	0	2045	1742	1465

alkane 1-monooxygenase [Acinetobacter sp. ANC 5318]	0	0	0	1841	1673	1350
antibiotic biosynthesis monooxygenase [Xanthobacter sp. 91]	0	0	0	1558	1567	1684
antibiotic biosynthesis monooxygenase [Xanthobacter sp. 91]	0	0	0	1340	1316	1467
antibiotic biosynthesis monooxygenase [Xanthobacter sp. 126]	0	0	0	1317	1286	1419
putative antibiotic biosynthesis monooxygenase [Acinetobacter baumannii 1106579]	0	0	0	1009	631	426
4-hydroxyphenylacetate 3-monooxygenase, oxygenase component [Bacillus novalis]	0	0	0	0	291	73
3-hydroxybenzoate 4-monooxygenase [Microbacterium sp. KCTC 39802]	0	0	0	0	38	310
Phenylalanine 4-monooxygenase [Bacillus cereus BDRD-ST24] [Bacillus cereus]	0	0	0	150	90	0
Flavin-dependent monooxygenase, reductase subunit HsaB [Arthrobacter saudimassiliensis]	0	0	0	0	25	250
nitronate monooxygenase [Bacillus sp. FJAT-29814]	0	0	0	0	195	37
nitronate monooxygenase [Bacillus sp. FJAT-29814]	0	0	0	0	180	47
nitronate monooxygenase [Bacillus thermocopriae]	0	0	0	0	174	51
FAD-binding monooxygenase, PheA/TfdB [Bacillus cereus AH676]	0	0	0	139	72	0
3-hydroxybenzoate 4-monooxygenase [Microbacterium sp. ST-M6]	0	0	0	0	29	218
nitronate monooxygenase [Bacillus sp. FJAT-29814]	0	0	0	0	169	32
nitronate monooxygenase [Bacillus fumarioli]	0	0	0	0	149	57
4-hydroxybenzoate 3-monooxygenase [Microbacterium aerolatum]	0	0	0	0	15	220
lactate 2-monooxygenase [Microbacterium trichothecenolyticum]	0	0	0	0	14	214
alkanesulfonate monooxygenase [Bacillus thuringiensis BMB171]	0	0	0	103	83	0

Table S4. The betweenness centrality value of each node in the co-occurrence network.

Node	BC value	Node	BC value
OTU001	301.15	OTU044	0.00
OTU002	316.86	OTU046	0.00
OTU003	137.96	OTU047	104.00
OTU004	153.50	OTU048	0.00
OTU005	0.00	OTU051	264.74
OTU006	1.00	OTU052	145.14
OTU007	161.56	OTU053	0.00
OTU008	199.27	OTU054	130.17
OTU009	105.00	OTU055	93.91
OTU010	0.00	OTU058	0.50
OTU011	76.15	OTU061	118.75
OTU012	0.50	OTU062	16.85
OTU013	0.00	OTU064	0.00
OTU014	0.00	OTU067	3.50
OTU015	208.84	OTU068	2.87
OTU016	44.35	OTU069	0.00
OTU017	0.00	OTU070	2.00
OTU018	0.00	OTU071	53.00

OTU019	53.00	OTU072	0.00
OTU020	0.00	OTU073	3.44
OTU021	3.06	OTU075	0.00
OTU023	0.00	OTU076	8.71
OTU024	28.45	OTU077	0.00
OTU025	27.28	OTU078	53.00
OTU026	160.47	OTU079	27.40
OTU027	1.14	OTU080	0.00
OTU028	0.00	OTU081	0.00
OTU030	54.41	OTU083	0.00
OTU031	0.00	OTU086	0.00
OTU032	2.99	OTU087	125.97
OTU033	0.00	OTU090	1.00
OTU034	0.00	OTU091	2.31
OTU035	58.73	OTU094	53.00
OTU038	0.00	OTU096	251.50
OTU039	0.00	OTU099	158.33
OTU040	6.92	OTU101	0.00
OTU041	79.08	OTU103	19.21
OTU042	0.00	OTU105	200.00

OTU043	0.00	OTU115	0.00

Table S5. Aldehyde dehydrogenase encoded genes that potentially involved in 1,4-dioxane degradation in metagenome data. Only the genes that exhibited significant differences between groups (*P* < 0.05 for Deseq2) and were present in at least two samples were shown.

Description	E1	E2	E3	F1	F2	F3
aldehyde dehydrogenase [Ochrobactrum anthropi]	3553	9146	6553	0	425	0
aldehyde dehydrogenase family protein [Shinella granuli]	4387	5949	7240	0	543	1
aldehyde dehydrogenase [Rhizobium sp. ACO-34A]	4411	6151	6783	0	538	0
aldehyde dehydrogenase family protein [Shinella granuli]	4135	5918	6476	0	503	0
aldehyde dehydrogenase (NADP(+)) [Shinella sp. HZN7]	4251	5817	6384	0	491	0
aldehyde dehydrogenase family protein [Ensifer aridi]	3747	6212	5330	0	450	0
aldehyde dehydrogenase [Shinella sp. HZN7]	1763	2486	2711	0	221	0
acetaldehyde dehydrogenase [Shinella sp. HZN7]	1209	1684	1939	0	153	0
aldehyde dehydrogenase family protein [Shinella sp. JR1-6]	130	1527	1279	0	0	0
aldehyde dehydrogenase [Oligotropha carboxidovorans]	268	728	227	0	0	0
aldehyde dehydrogenase family protein [Oligotropha carboxidovorans]	256	631	172	0	0	0
aldehyde dehydrogenase family protein [Oligotropha carboxidovorans]	182	598	162	0	0	0
aldehyde dehydrogenase family protein [Lacibacter cauensis]	0	397	449	0	0	0
betaine-aldehyde dehydrogenase [Sphingobacteriales bacterium 46-32]	0	335	452	0	0	0

aldehyde dehydrogenase (NADP(+)) [Sphingobacteriales bacterium 46-32]	0	327	432	0	0	0
aldehyde dehydrogenase [Sphingobacteriales bacterium 46-32]	0	356	401	0	0	0
aldehyde dehydrogenase family protein [Sphingobacteriales bacterium 46-32]	0	341	412	0	0	0
aldehyde dehydrogenase [Sphingobacteriales bacterium SCN 48-20]	0	297	401	0	0	0
aldehyde dehydrogenase [Sphingobacteriales bacterium 46-32]	0	225	257	0	0	0
aldehyde dehydrogenase [Sphingobacteriales bacterium 46-32]	0	76	114	0	0	0
aldehyde dehydrogenase family protein [Deinococcus aquatilis]	62	56	17	0	0	0
aldehyde dehydrogenase family protein [Deinococcus sp. H1]	64	41	9	0	0	0
aldehyde dehydrogenase family protein [Deinococcus aquatilis]	52	33	7	0	0	0
aldehyde dehydrogenase [Nocardia nova]	55	3	0	2	0	0
aldehyde dehydrogenase family protein [Leifsonia sp. ku-ls]	42	0	0	0	0	0
aldehyde dehydrogenase family protein [Ochrobactrum tritici]	23	0	0	0	0	0
acetaldehyde dehydrogenase (acetylating) [Bacillus abyssalis]	0	0	0	0	139	27
aldehyde dehydrogenase [Bacillus cereus]	0	0	0	75	94	0
aldehyde dehydrogenase family protein [Microbacterium sp. Root166]	0	0	0	0	19	174
aldehyde dehydrogenase family protein [Microbacterium sp. Root166]	0	0	0	0	20	178
Aldehyde dehydrogenase [Bacillus cereus ATCC 14579] [Bacillus cereus]	0	0	0	129	84	0
aldehyde dehydrogenase family protein [Acinetobacter sp. CIP 102136]	0	0	0	105	83	55
aldehyde dehydrogenase (NAD+) [Acidovorax sp. SLBN-42]	0	0	0	0	15	234
aldehyde dehydrogenase family protein [Pseudarthrobacter sp. ATCC 49987]	0	0	0	0	15	238

aldehyde dehydrogenase [Sinomonas sp. R1AF57]	0	0	0	0	13	256
aldehyde dehydrogenase family protein [Microbacterium sp. SLBN-154]	0	0	0	0	25	276
aldehyde dehydrogenase family protein [Bacillus sp. FJAT-14578]	0	0	0	0	234	73
aldehyde dehydrogenase family protein [Microbacterium sp. LAM7116]	0	0	0	0	22	289
aldehyde dehydrogenase family protein [Ammoniphilus sp. YIM 78166]	0	0	0	0	259	57
aldehyde dehydrogenase family protein [Bacillus niacini]	0	0	0	0	268	49
aldehyde dehydrogenase [Bacillus sp. FJAT-29814]	0	0	0	0	266	63
aldehyde dehydrogenase family protein [Bacillus rubiinfantis]	0	0	0	0	276	58
aldehyde dehydrogenase family protein [Psychrobacillus soli]	0	0	0	0	257	78
aldehyde dehydrogenase family protein [Bacillus dakarensis]	0	0	0	0	287	55
aldehyde dehydrogenase family protein [Bacillus pseudofirmus]	0	0	0	0	296	53
aldehyde dehydrogenase family protein [Bacillus sp. FJAT-29814]	0	0	0	0	288	82
aldehyde dehydrogenase [Acinetobacter sp. ANC 5347]	0	0	0	190	125	94
aldehyde dehydrogenase family protein [Acinetobacter sp. 18QD2AZ41W]	0	0	0	1407	712	513
acetaldehyde dehydrogenase (acetylating) [Acinetobacter sp. RIT592]	0	0	0	1407	1206	1042
aldehyde dehydrogenase [Acinetobacter sp. CIP 51.11]	0	0	0	1652	1372	1115
aldehyde dehydrogenase family protein [Acinetobacter lwoffii]	0	0	0	2457	1926	1722
aldehyde dehydrogenase family protein [Acinetobacter sp. AR2-3]	0	0	0	2479	1932	1798
betaine aldehyde dehydrogenase [Acinetobacter sp. CIP 64.7]	0	0	0	2562	2045	1850
coniferyl aldehyde dehydrogenase [Acinetobacter lwoffii]	0	0	0	2603	2123	1974

aldehyde dehydrogenase iron-sulfur subunit [Xanthobacter sp. 91]	0	0	0	2726	2651	3107
aldehyde dehydrogenase family protein [Xanthobacter sp. 91]	0	0	0	5782	5632	6636
aldehyde dehydrogenase family protein [Xanthobacter sp. 126]	0	0	0	6782	6551	7299
aldehyde dehydrogenase family protein [Mesorhizobium sp. GR13]	0	0	0	6180	6483	8411
aldehyde dehydrogenase [Xanthobacter sp. 91]	0	0	0	6987	6700	7797
aldehyde dehydrogenase [Xanthobacter sp. 126]	0	0	0	6984	6800	7785
aldehyde dehydrogenase [Xanthobacter tagetidis]	0	0	0	7098	6905	7724
aldehyde dehydrogenase family protein [Mesorhizobium sp. GR13]	0	0	0	6528	6663	9062

Table S6. Alcohol dehydrogenase encoded genes that potentially involved in 1,4-dioxane degradation in metagenome data. Only the genes that exhibited significant differences between groups (*P* < 0.05 for Deseq2) and were present in at least two samples were shown.

Description	E1	E2	E3	F1	F2	F3
class III alcohol dehydrogenase [Shinella zoogloeoides]	3503	5021	5591	0	421	0
alcohol dehydrogenase [Shinella sp. HZN7]	2715	4643	4967	0	327	0
iron-containing alcohol dehydrogenase [Shinella granuli]	2869	4056	4653	0	375	0
iron-containing alcohol dehydrogenase [Shinella granuli]	3042	3977	4402	0	390	0
zinc-binding alcohol dehydrogenase family protein [Shinella sp. JR1-6]	2518	4058	3551	1	313	0
iron-containing alcohol dehydrogenase [Sinorhizobium sp. RAC02]	2509	3891	3516	0	325	0
NAD(P)-dependent dehydrogenase [Shinella granuli]	2271	3354	3813	0	289	0
zinc-dependent alcohol dehydrogenase family protein [Shinella granuli]	2032	2859	3307	0	273	0
alcohol dehydrogenase catalytic domain-containing protein [Shinella kummerowiae]	224	4073	2876	0	0	0
NAD(P)-dependent alcohol dehydrogenase [Shinella sp. HZN7]	1615	2981	2434	0	184	0
NAD(P)-dependent alcohol dehydrogenase [Shinella sp. HZN7]	1737	2292	1909	0	204	0
zinc-dependent alcohol dehydrogenase family protein [Shinella zoogloeoides]	169	2831	2373	0	0	0
NAD(P)-dependent dehydrogenase [Shinella granuli]	1499	1615	1855	0	162	0
short-chain alcohol dehydrogenase [Rhizobium sp. AP16]	105	1550	1423	0	0	0
class III alcohol dehydrogenase [Oligotropha carboxidovorans]	212	515	147	0	0	0

Zn-dependent alcohol dehydrogenase [Oligotropha carboxidovorans]	240	490	137	0	0	0
zinc-binding alcohol dehydrogenase family protein [Oligotropha carboxidovorans]	178	443	127	0	0	0
NAD(P)-dependent alcohol dehydrogenase [Oligotropha carboxidovorans]	192	418	133	0	0	0
zinc-dependent alcohol dehydrogenase family protein [Oligotropha carboxidovorans]	158	474	109	0	0	0
alcohol dehydrogenase catalytic domain-containing protein [Oligotropha carboxidovorans]	208	435	97	0	0	0
zinc-binding alcohol dehydrogenase [Oligotropha carboxidovorans OM5]	168	417	142	0	0	0
NAD(P)-dependent alcohol dehydrogenase [Gynurincola endophyticus]	0	311	310	0	0	0
alcohol dehydrogenase [Sphingobacteriales bacterium 46-32] [Sphingobacteriales bacterium]	0	286	325	0	0	0
iron-containing alcohol dehydrogenase [Deinococcus aquatilis]	53	40	10	0	0	0
zinc-dependent alcohol dehydrogenase family protein [Deinococcus aquatilis]	37	31	7	0	0	0
NAD(P)-dependent alcohol dehydrogenase [Deinococcus aquatilis]	39	25	7	0	0	0
alcohol dehydrogenase catalytic domain-containing protein [Deinococcus sp. Arct2-2]	41	15	13	0	0	0
iron-containing alcohol dehydrogenase [Meiothermus granaticius]	40	21	8	0	0	0
NAD(P)-dependent alcohol dehydrogenase [Deinococcus aquatilis]	31	22	1	0	0	0
Zn-dependent alcohol dehydrogenase [Leifsonia sp. ku-ls]	33	0	0	0	0	1
alcohol dehydrogenase [Fusarium oxysporum f. sp. vasinfectum 25433]	18	0	0	0	0	0
Alcohol dehydrogenase, zinc-containing [Bacillus cereus BDRD-ST24]	0	0	0	57	67	3
NAD(P)-dependent alcohol dehydrogenase [Microbacterium trichothecenolyticum]	0	0	0	0	18	175
zinc-dependent alcohol dehydrogenase family protein [Bacillus bataviensis]	0	0	0	0	168	38
NAD(P)-dependent alcohol dehydrogenase [Bacillus sp. AFS031507]	0	0	0	0	156	53
alcohol dehydrogenase catalytic domain-containing protein [Paenibacillus sp. UNC451MF]	0	0	0	0	187	30
zinc-dependent alcohol dehydrogenase family protein [Bacillus massilionigeriensis]	0	0	0	0	197	37
iron-containing alcohol dehydrogenase [Bacillus sp. FJAT-29814]	0	0	0	0	201	34
alcohol dehydrogenase catalytic containing protein [Rhodobacteraceae bacterium WD3A24]	0	0	0	0	214	31
alcohol dehydrogenase catalytic domain-containing protein [Bacillus sp. MM2020_4]	0	0	0	0	192	53
NAD(P)-dependent alcohol dehydrogenase [Cohnella sp. 6021052837]	0	0	0	0	213	35
iron-containing alcohol dehydrogenase [Bacillus sp. FJAT-29814]	0	0	0	0	223	30
iron-containing alcohol dehydrogenase [Bacillus persicus]	0	0	0	0	223	35
alcohol dehydrogenase catalytic domain-containing protein [Bacillus vireti]	0	0	0	0	199	63
iron-containing alcohol dehydrogenase [Bacillus sp. FJAT-29814]	0	0	0	0	217	71
iron-containing alcohol dehydrogenase [Bacillus sp. X1(2014)]	0	0	0	0	241	50
iron-containing alcohol dehydrogenase [Bacillus sp. FJAT-29814]	0	0	0	0	259	69
Alcohol dehydrogenase, iron-containing [Bacillus cereus BDRD-ST24]	0	0	0	221	146	2
bifunctional acetaldehyde-CoA/alcohol dehydrogenase [Bacillus sp. FJAT-29814]	0	0	0	0	491	106
bifunctional acetaldehyde-CoA/alcohol dehydrogenase [Bacillus sp. FJAT-29814]	0	0	0	0	477	122
NAD(P)-dependent dehydrogenase [Rhizobiales bacterium GAS113]	0	0	0	0	422	545
alcohol dehydrogenase catalytic domain-containing protein [Acinetobacter lwoffii]	0	0	0	1862	1421	1229
alcohol dehydrogenase [Acinetobacter sp. HA]	0	0	0	1870	1387	1274
class III alcohol dehydrogenase [Acinetobacter sp. CIP 51.11]	0	0	0	1857	1548	1390
zinc-dependent alcohol dehydrogenase family protein [Acinetobacter lwoffii]	0	0	0	2384	1519	1078
NAD(P)-dependent alcohol dehydrogenase [Acinetobacter sp. CIP 101966]	0	0	0	2072	1600	1349

iron-containing alcohol dehydrogenase [Acinetobacter lwoffii]	0	0	0	2027	1616	1443
alcohol dehydrogenase, iron-dependent [Acinetobacter lwoffii SH145]	0	0	0	1943	1735	1443
putative alcohol dehydrogenase, zinc-containing [Acinetobacter seifertii]	0	0	0	2994	2032	1411
iron-containing alcohol dehydrogenase [Acinetobacter indicus]	0	0	0	3343	2239	1461
alcohol dehydrogenase [Acinetobacter lwoffii]	0	0	0	3538	2237	1581
zinc-dependent alcohol dehydrogenase family protein [Xanthobacter sp. 91]	0	0	2	4389	4219	4869
zinc-binding alcohol dehydrogenase [Xanthobacter sp. 126]	0	0	0	4421	4258	4847
alcohol dehydrogenase catalytic domain-containing protein [Xanthobacter sp. 91]	0	0	0	4557	4527	4989
zinc-binding alcohol dehydrogenase family protein [Xanthobacter sp. 91]	0	0	0	4725	4553	5305
Zn-dependent alcohol dehydrogenase [Xanthobacter sp. 91]	0	0	0	5047	5191	5556
alcohol dehydrogenase catalytic domain-containing protein [Xanthobacter sp. 126]	0	0	0	5448	5105	5805
NAD(P)-dependent alcohol dehydrogenase [Xanthobacter sp. 91]	0	0	0	5528	5029	5926

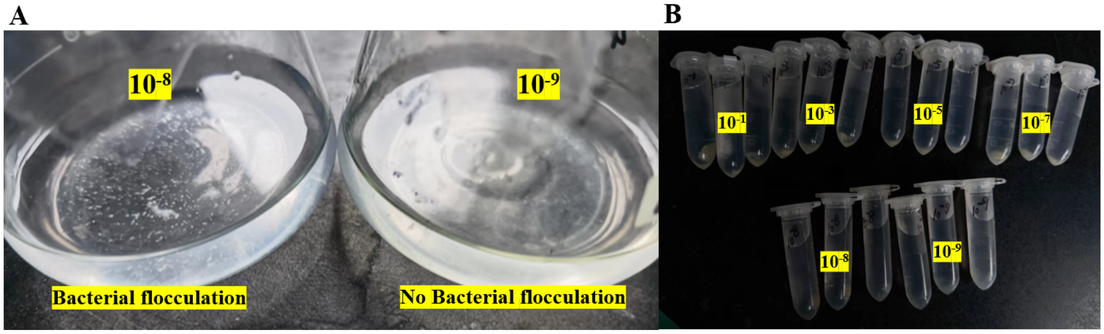

Figure S1 Phenotype of microbial consortia growth in liquid medium, a comparison of 10^{-8} and 10^{-9} (**A**). Transfer 2 mL of bacterial solution from 10^{-1} to 10^{-9} to 2 mL centrifuge tubes for natural sedimentation (**B**).

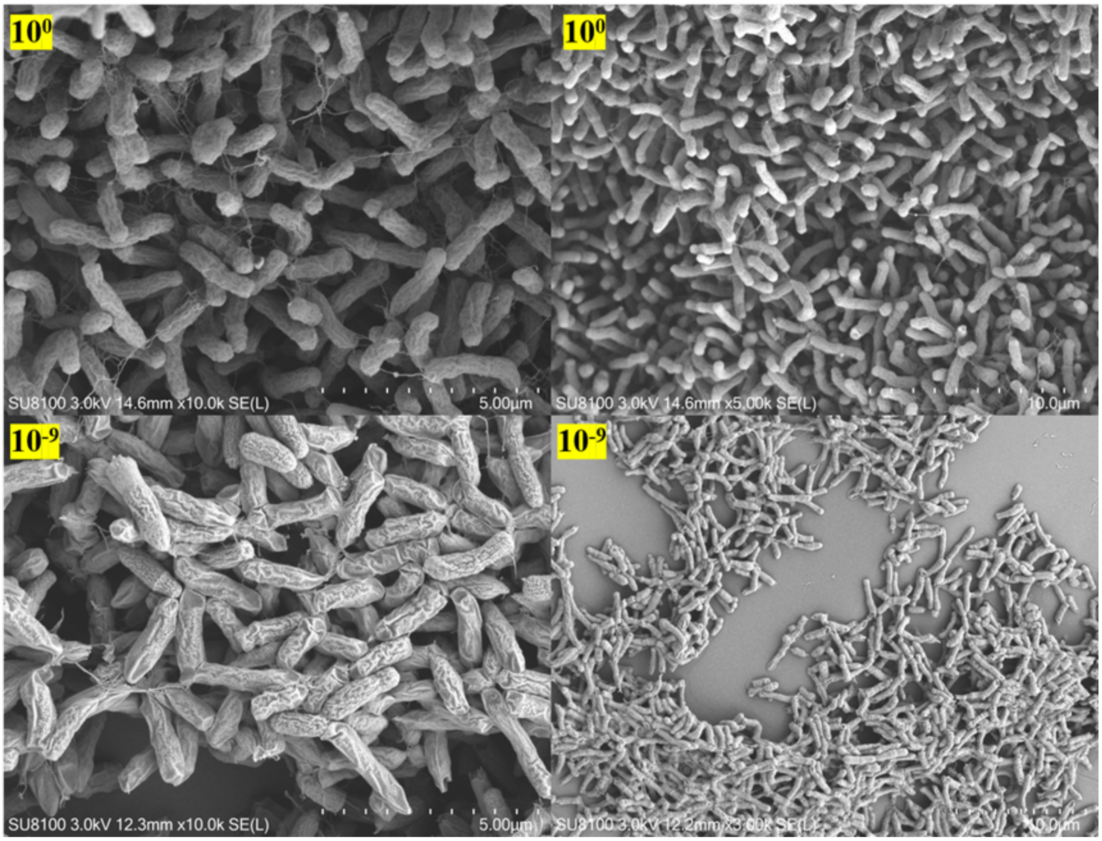

Figure S2 Scanning electron microscopy (SEM) of the original microbial consortium and the 10^{-9} microbial consortium.

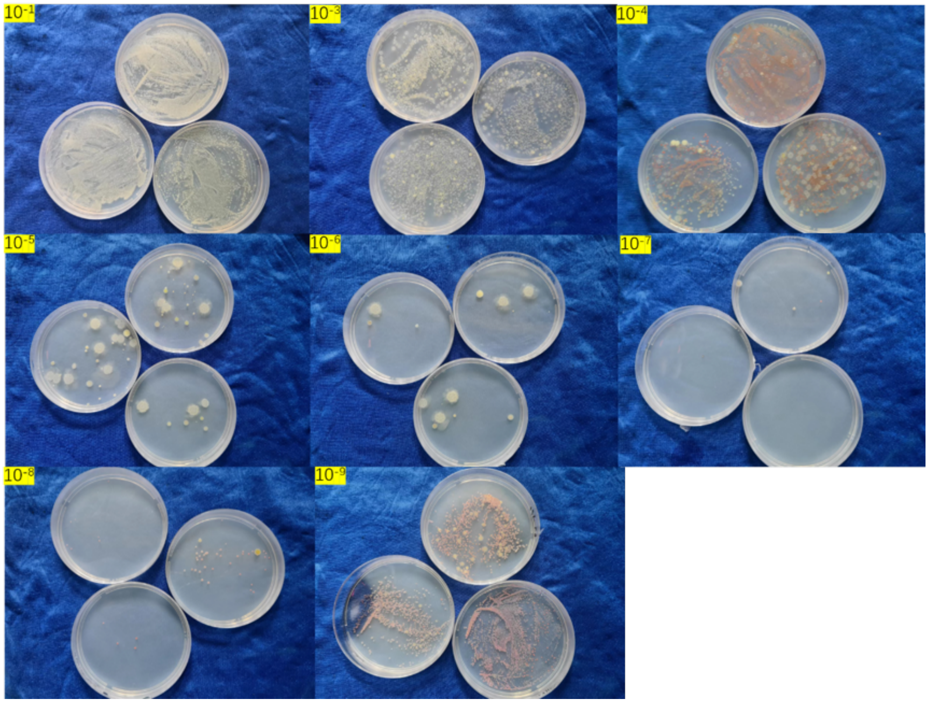

Figure S3 Growth colonies after dilution and coating on R2A plates. (10^{-1}, 10^{-3}, 10^{-4}, 10^{-5}, 10^{-6}, 10^{-7}, 10^{-8}, 10^{-9}).

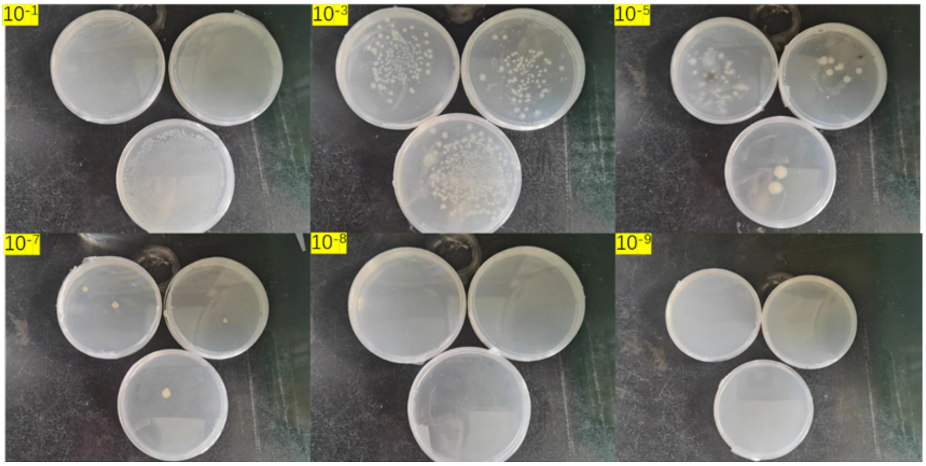

Figure S4 Growth colonies after dilution and coating on 1,4-dioxane plates. (10^{-1}, 10^{-3}, 10^{-5}, 10^{-7}, 10^{-8}, 10^{-9}).

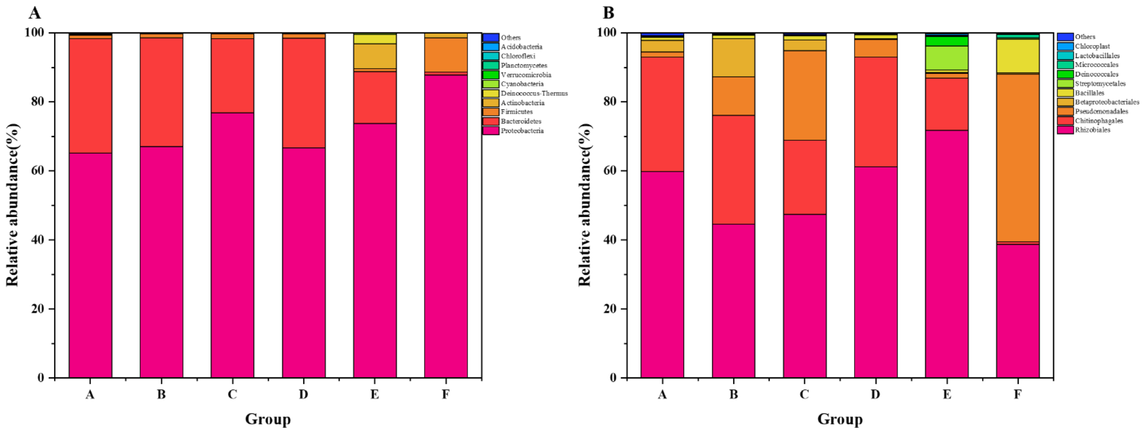

Figure S5 Stacked bar charts of the most abundant 10 tax at the phylum (**A**) and order (**B**) level. The rest are classified as Others. The group A to E represents the dilution factor of 10^0 to 10^{-9}.

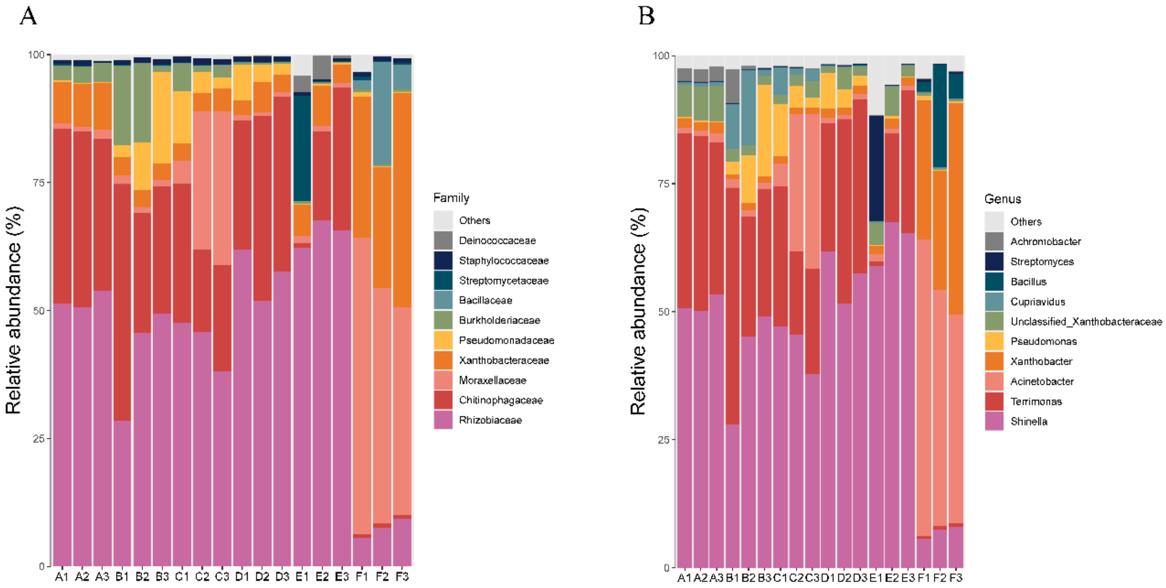

Figure S6 Stacked bar charts of the most abundant 10 tax at the family level (**A**) and genus level (**B**). The rest are classified as Others. The group A to E represents the dilution factor of 10^0 to 10^{-9}.

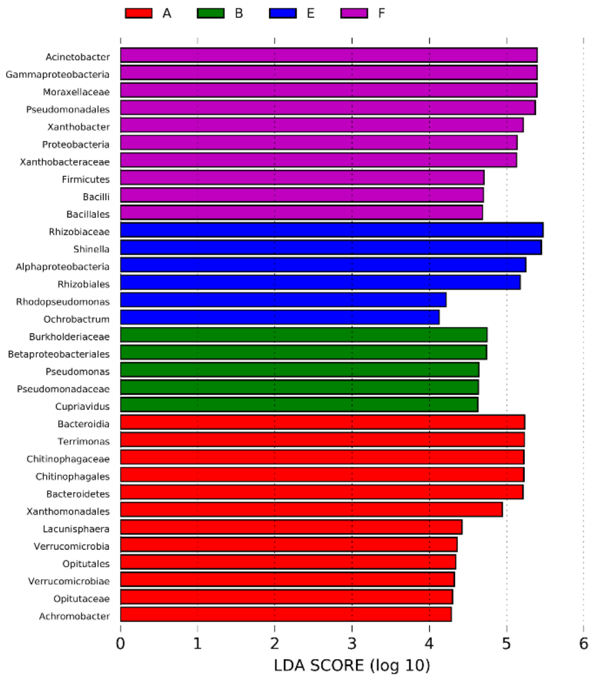

Figure S7 LEfSe (linear discriminant analysis effect size) analysis of the taxonomic differences among all groups. LDA score threshold > 4, and $P < 0.05$ were considered statistically significant.

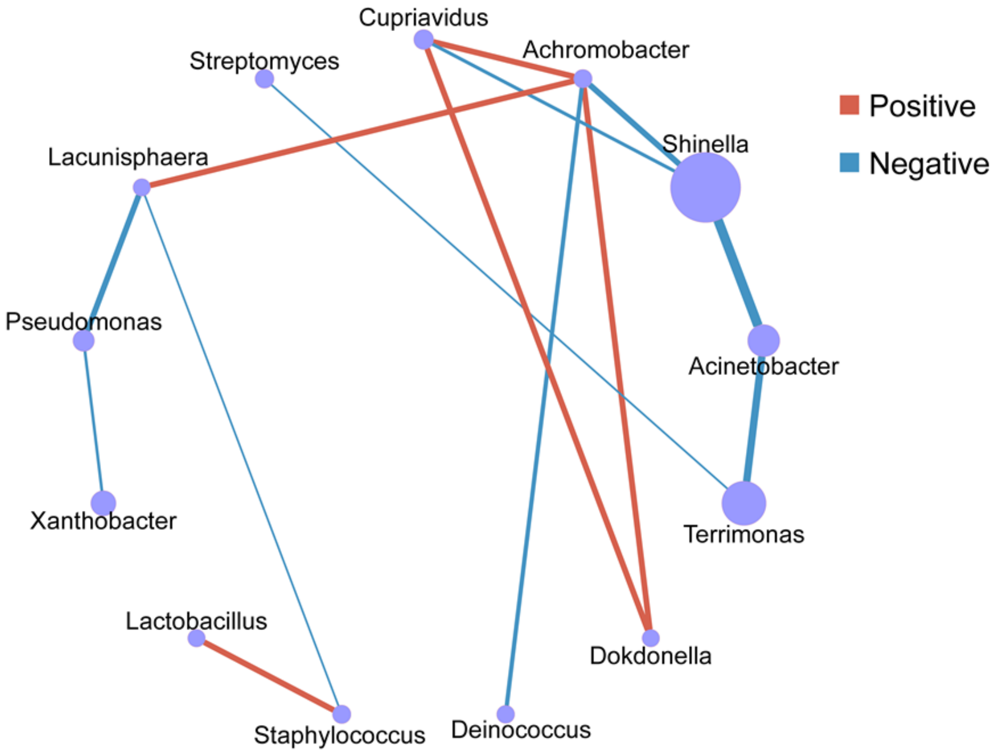

Figure S8 Co-occurrence network based on spearman's correlation at the genus level. The size of the nodes represents the relative abundance of the genus. The size of the edge represents the strength of the correlation coefficient. Correlation coefficient |r| > 0.6 and |*P*| < 0.05 was used as the threshold.

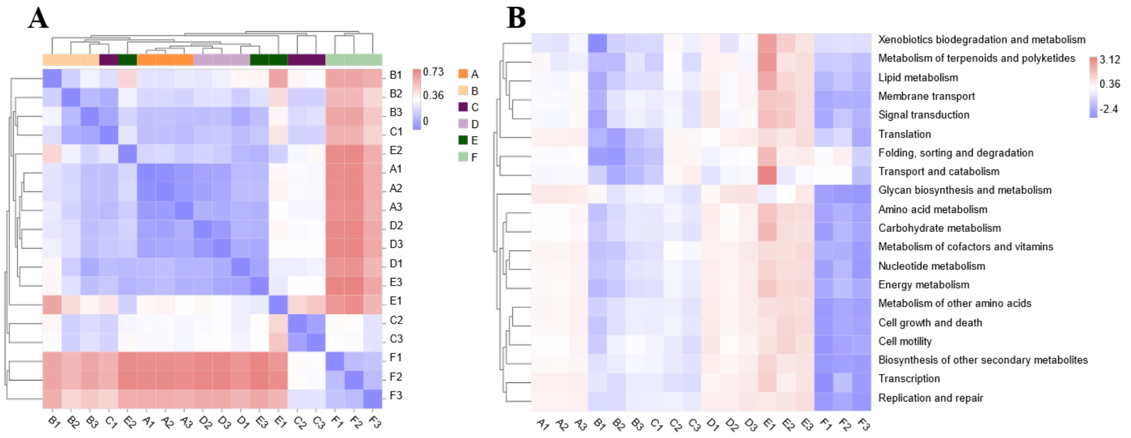

Figure S9 Heat maps of weighted UniFrac distance matrices (**A**). PICRUSt2 function prediction profile level2 abundance heatmap (**B**). A, B, C, D, E, and F was equivalent to dilution factor of 10^0, 10^{-3}, 10^{-5}, 10^{-7}, 10^{-8}, 10^{-9}.

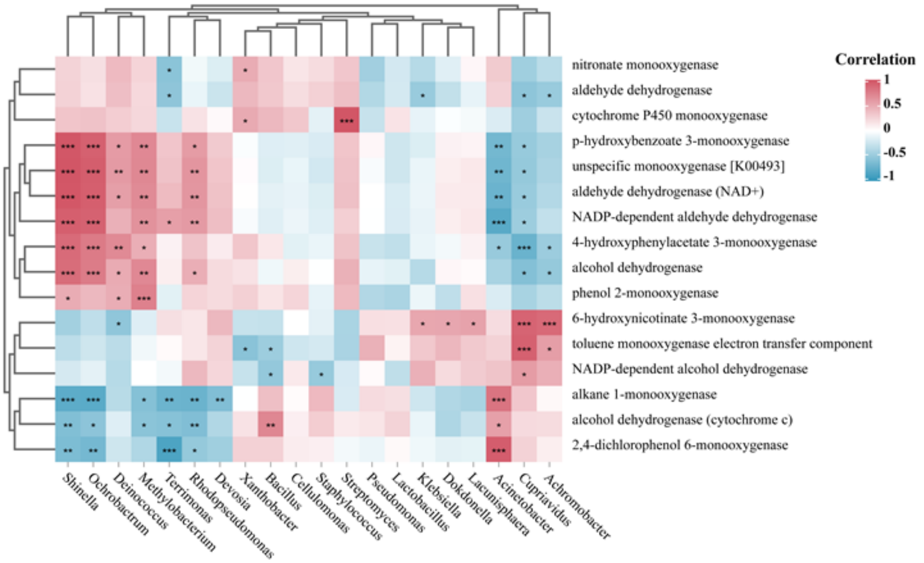

Figure S10 Correlation between species and function under series dilution process. * indicates $P < 0.05$, ** indicates $P < 0.01$, and*** indicates $P < 0.001$. The Spearman's rank correlation coefficient was calculated using the copy numbers of key function-coding genes and the abundance of the top 20 genera.

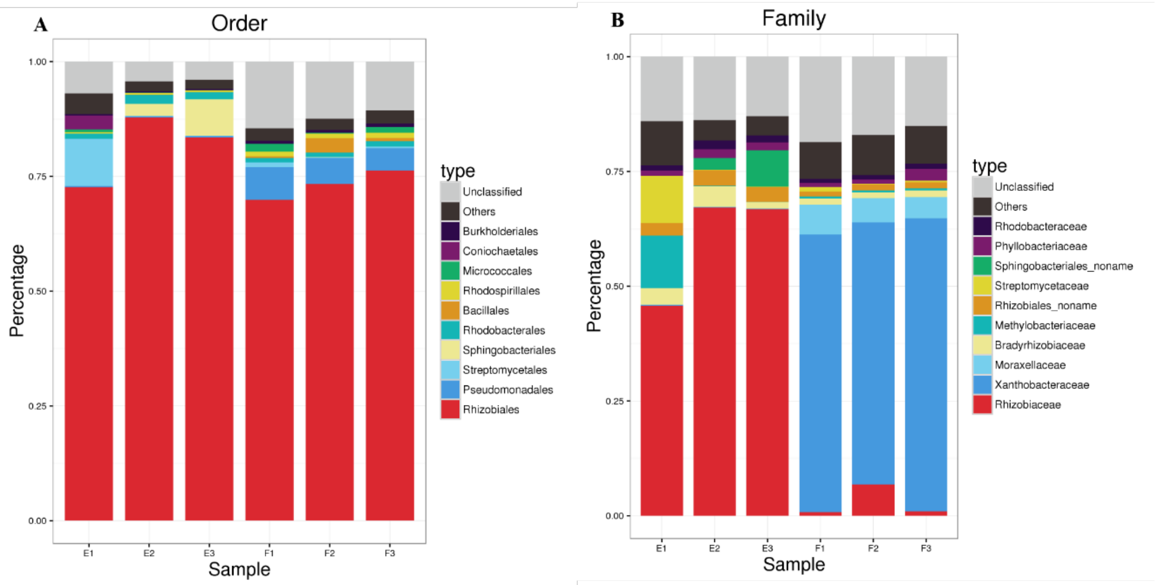

Figure S11 Stacked bar charts of the most abundant 10 tax by metagenome analysis at the order (**A**) and family (**B**) level. The remaining are classified as Others and Unclassified. The group E and F represents

the dilution factor of 10^{-8} and 10^{-9}.

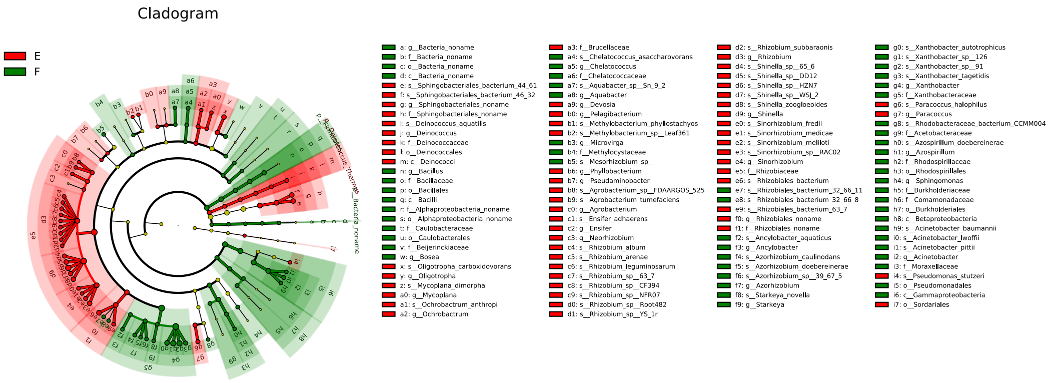

Figure S12 LEfSe (linear discriminant analysis effect size) analysis of the relative abundance of species in consortium E (10^{-8}) and consortium F (10^{-9}) annotated by metagenomic sequence data. LDA score threshold > 4, and $P < 0.05$ were considered statistically significant.

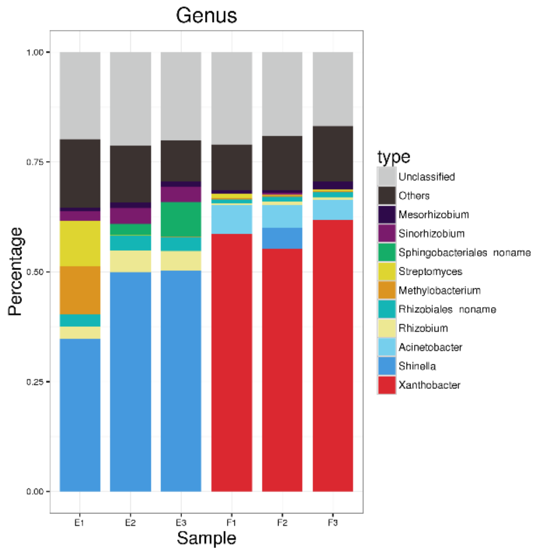

Figure S13 The top 10 predominant genus in metagenome data. The remaining was categorized to Others and Unclassified.

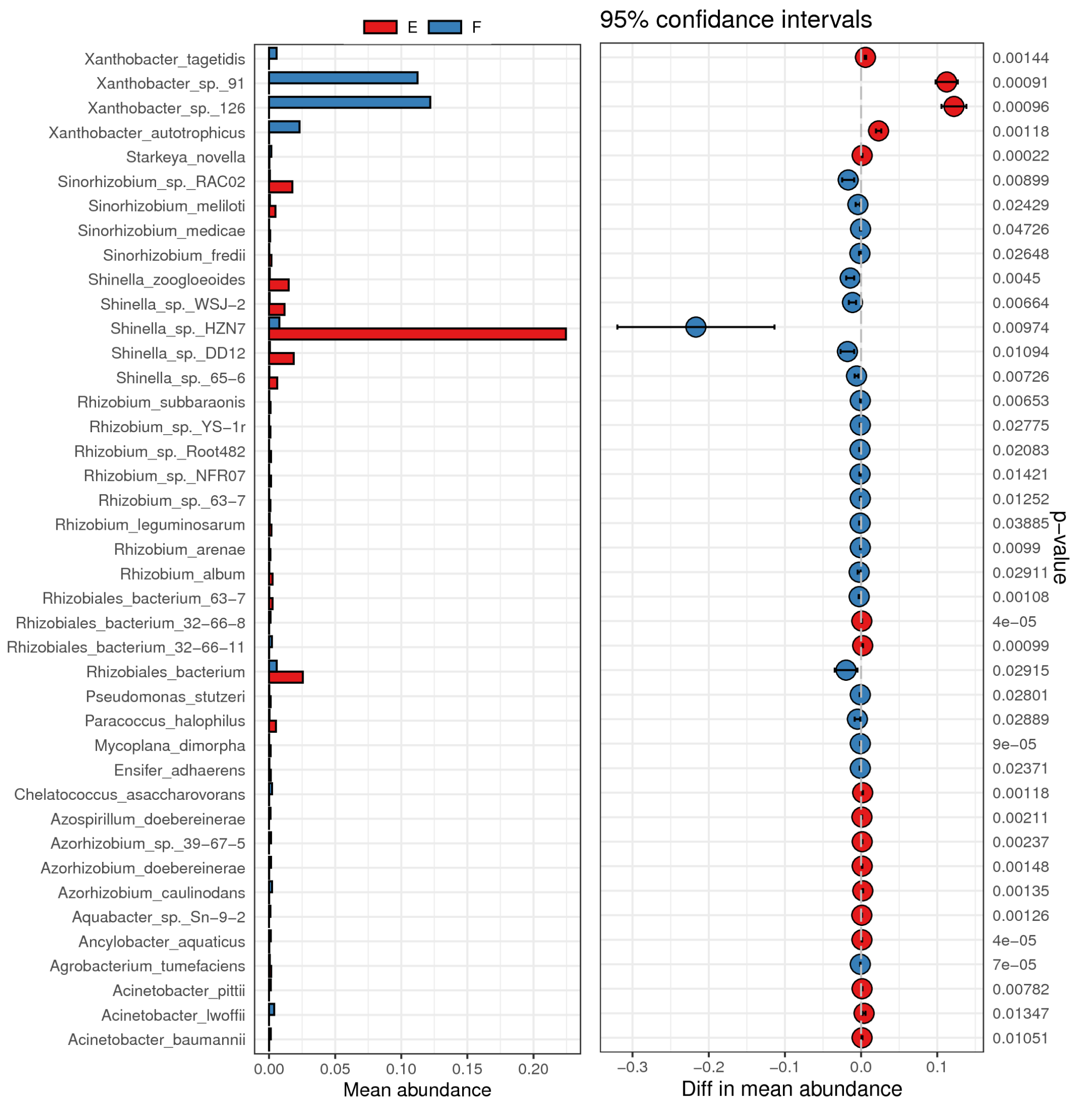

Figure S14 Welch's t-test bar plot reveals species with significant differences ($P < 0.05$) between 10^{-8} and 10^{-9}. The group mean of the relative abundance of species annotated in the metagenome are shown.

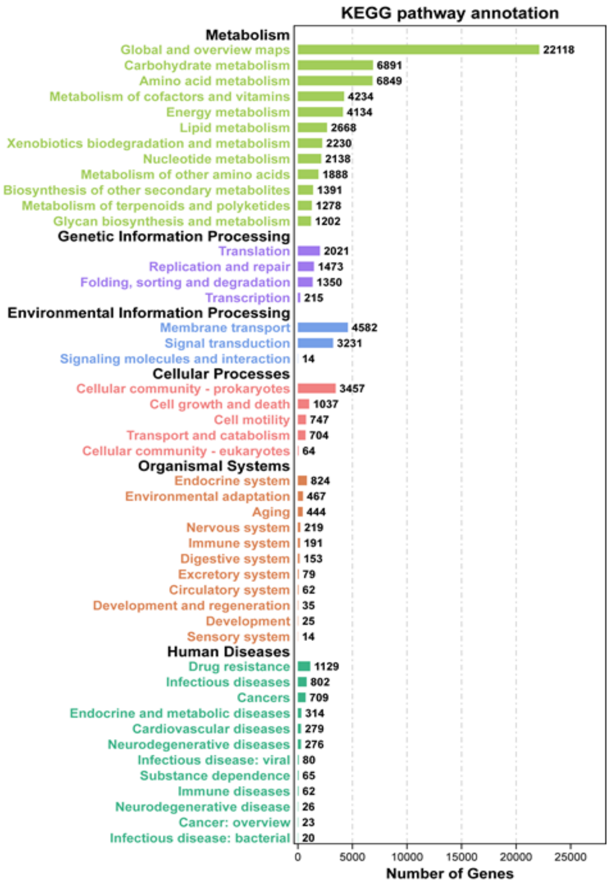

Figure S15 Number of genes (114333) assigned to KEGG pathway at Level1 and Level2.

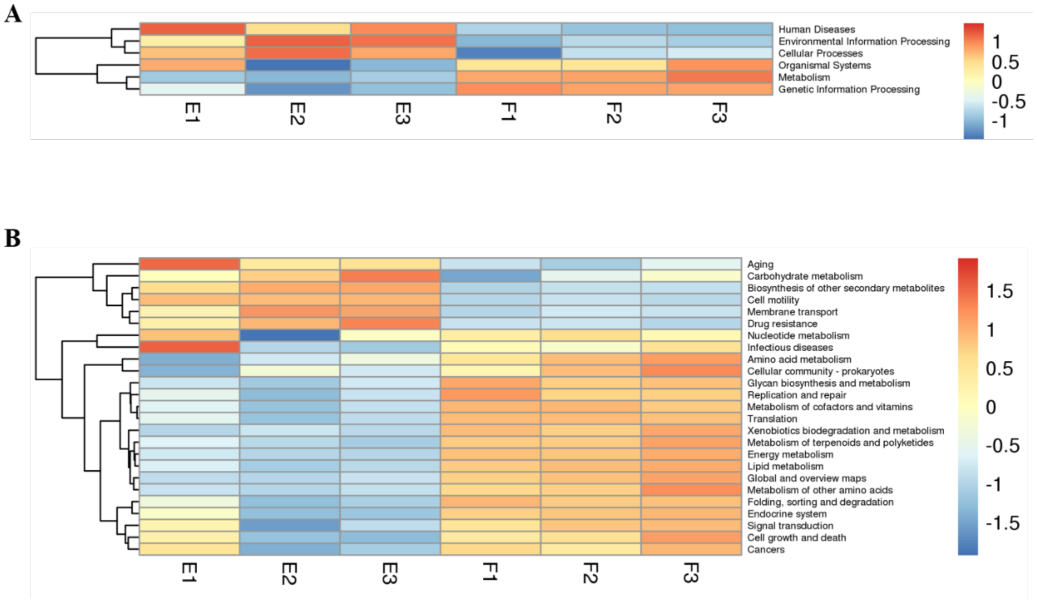

Figure S16 Heatmap of KEGG pathway level 1 (**A**) and 2 (**B**). Group E and F represent the dilution

factor of 10^{-8} and 10^{-9}.

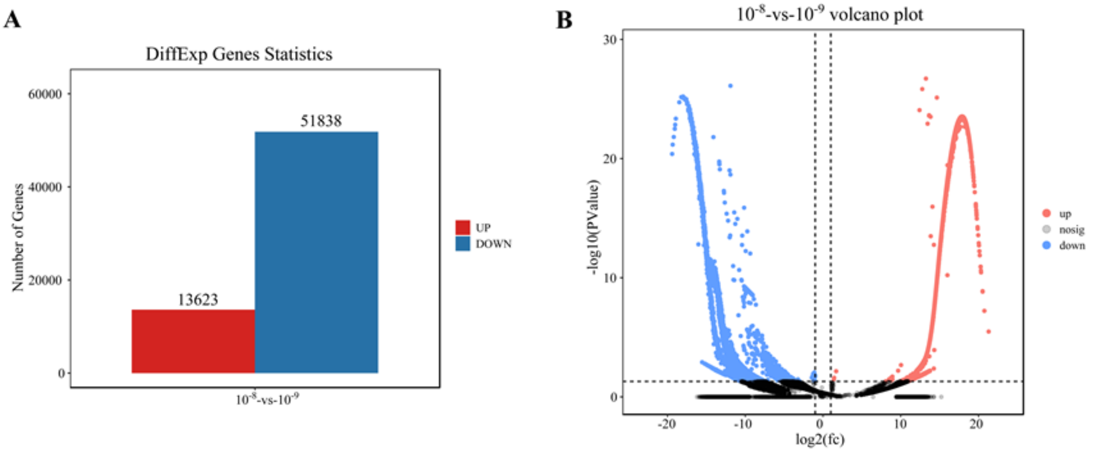

Figure S17 Differential gene expression results in 10^{-8} and 10^{-9} consortium (Deseq2 analysis). Differentially expressed genes (*P*. adj < 0.05) were identified as up- or down-regulated by the positive or negative log fold changes values.

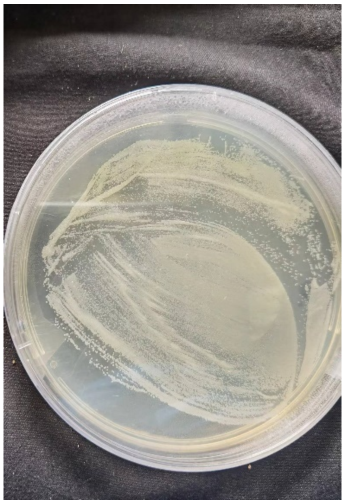

Figure S18 Pure culture colonies of isolate DXTK-001 (*Shinella* sp.) on 1,4-dioxane MSM agar plate.

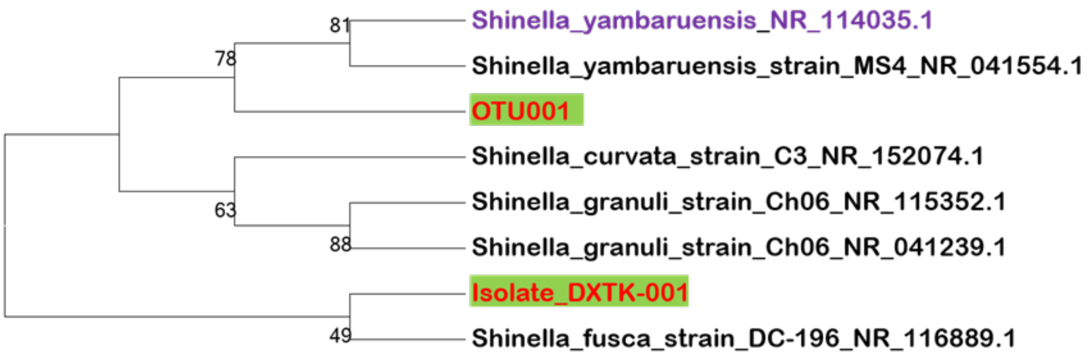

Figure S19 Neighbor-joining phylogenetic characterization of OTU001 and isolated DXTK-001. Reference taxa sequence was BLAST results with the highest identity scores to OTU001 and DXTK-001. Bootstrap values were generated after 1500 resampling using the MEGA.

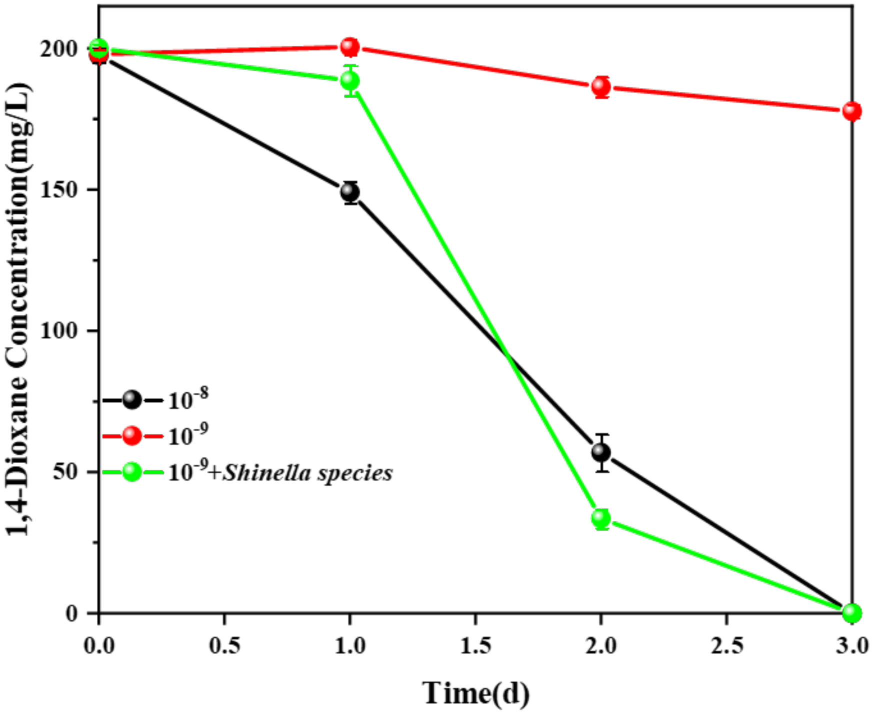

Figure S20 1,4-Dioxane degradation curves by 10^{-8}, 10^{-9} consortium and 10^{-9} with adding *Shinella* sp. (C_0= 200 mg L^{-1}, Initial OD_{600}= 0.1).

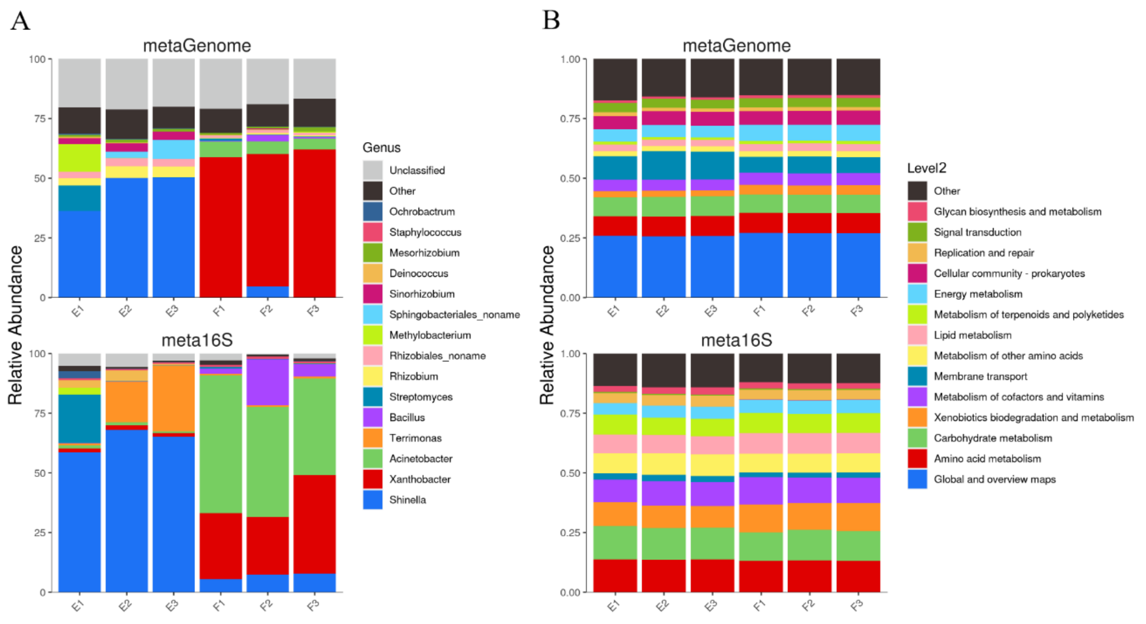

Figure S21 The top 10 species/features in metagenome and 16S rRNA sequences at genus level (**A**) and KEGG level2 (**B**), other known species/features are categorized as other, and unknown species/features are marked as unclassified.

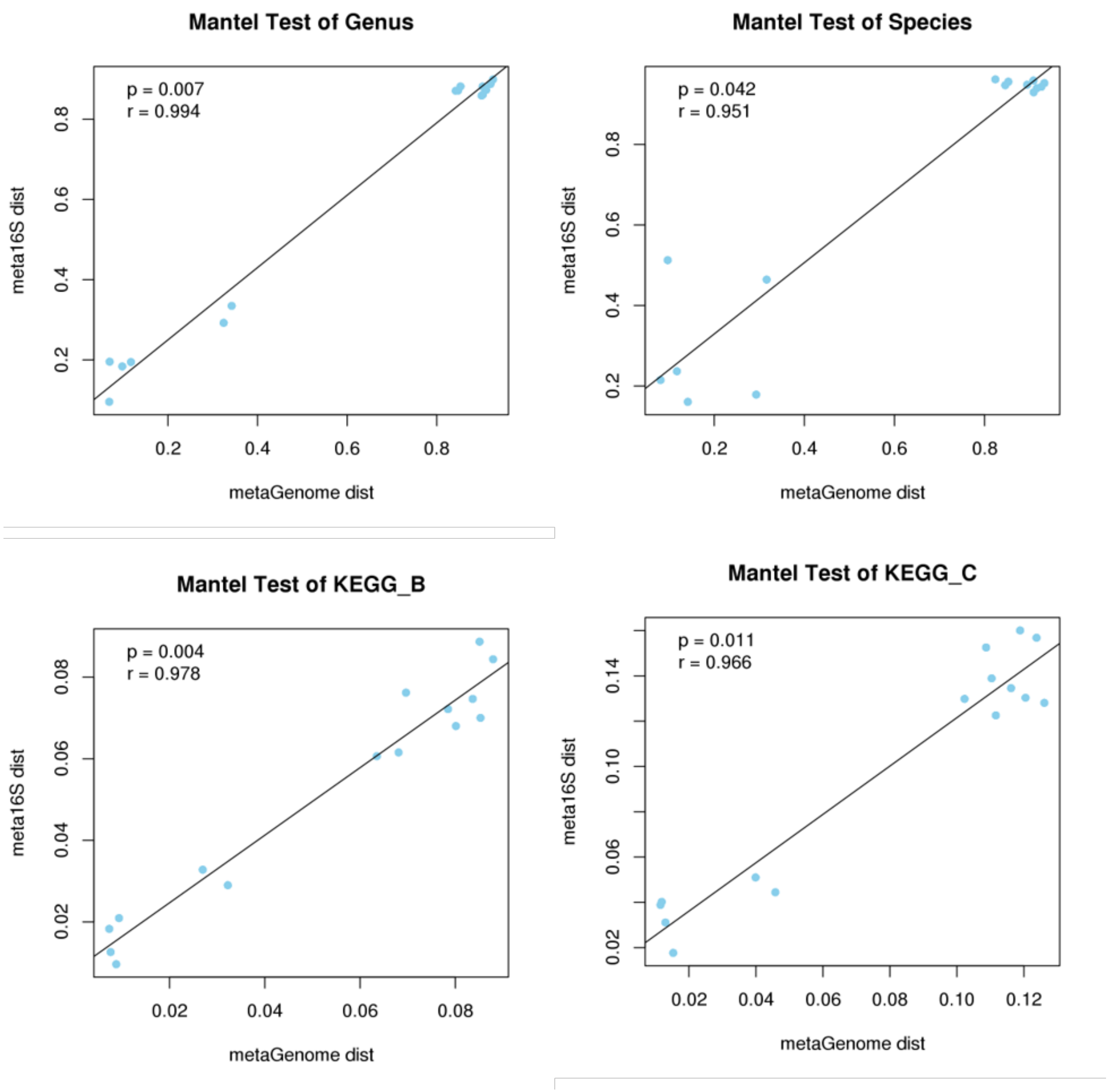

Figure S22 Mantel test (999 permutations) for the correlation of metagenomic and 16S rRNA sequences. The Bray-Curtis distance matrix was calculated based on the species/functional abundance tables obtained from 16S rRNA sequencing and metagenome sequencing, and then the correlation between the 16S rRNA and metagenome sequencing results was tested at genus & species level/KEGG level1 & KEGG level2.

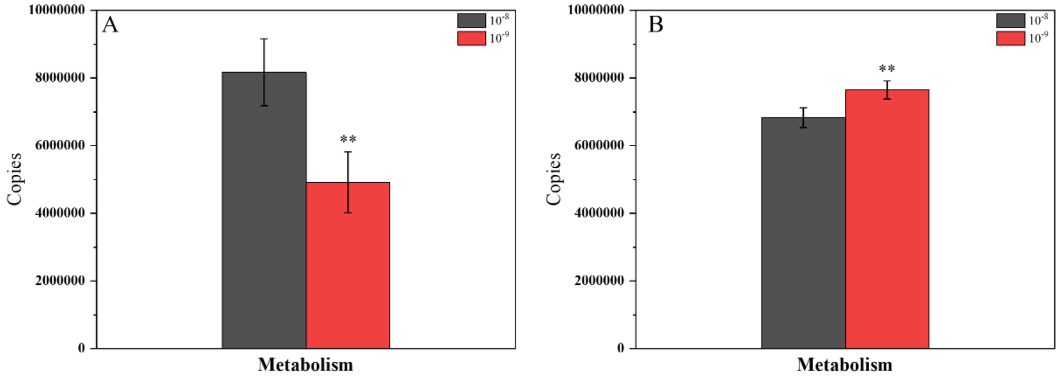

Figure S23 PICRUSt2 predicted profiles (**A**) and metagenome functional profiles (**B**) in Level 1 metabolism. Welch's t-test between 10^{-8} and 10^{-9}, ** $P < 0.01$.
